# In a UK sample, EMDR and other trauma therapists indicate beliefs in unconscious repression and dissociative amnesia

**DOI:** 10.1080/09658211.2025.2498929

**Published:** 2025-05-11

**Authors:** Pamela J. Radcliffe, Lawrence Patihis

**Affiliations:** School of Psychology, Sport and Health Sciences, University of Portsmouth, Portsmouth, UK

**Keywords:** Unconscious repression, dissociative amnesia, false memories, EMDR, trauma therapy

## Abstract

This study explored UK mental health professionals’ beliefs (*N* = 178) for autobiographical memory function for trauma in the context of adverse therapeutic outcomes, e.g., false memories. It captures novel data on controversial memory beliefs for unconscious repression, dissociative amnesia and dissociative identity disorder (DID). Study participants were mental health professionals and included non-trauma-focused, (*n* = 92), trauma-focused EMDR practitioners (*n* = 62) and (non-EMDR) trauma-focused practitioners (*n* = 24). Most study participants indicated some degree of belief in repression (>78%) and dissociative amnesia (>84%). EMDR and other trauma-focused practitioners showed elevated agreement for controversial memory notions. The EMDR practitioner group also showed more belief in the diagnostic validity of DID. New data on mental health professionals’ beliefs about the aetiology of psychogenic non-epileptic seizures (PNES) was also captured. Most study participants “Somewhat agreed” or “Agreed” that “blocked out” trauma memories are causally related to dissociation and physical symptoms, e.g., PNES (>78%); EMDR practitioners showed the highest degree of agreement (91%). The impact of memory beliefs alongside EMDR theory and practice is considered in the context of adverse therapeutic outcomes, e.g., false or non-experienced memories. Recommendations are made for future research to mitigate against adverse health outcomes.

Forgive no error you recognize, it will repeat itself, increase, and afterwards our pupils will not forgive in us what we forgave. Yevegny Yevtushenko (2001)

Some scholars have argued that modern-day mental health professionals’ beliefs for the controversial notions of unconscious repression and dissociative amnesia may again harm public health. In the past, such beliefs were associated with risky memory recovery techniques that elicited purported memories for non-experienced events (Loftus, 1993; Yapko, 1994a). Yet recent international research shows a sizable belief in unconscious repression persists amongst mental health professionals (Battista et al., 2023; Otgaar et al., 2019, 2021a; Patihis et al., 2014) along with problematic memory recovery techniques (Schemmel et al., 2024). Moreover, emerging research on belief in dissociative amnesia (a comparable notion) shows similar high beliefs amongst therapeutic professionals and lay persons (Otgaar et al., 2025a; Zappalà et al., 2024).

An important research gap is understanding UK mental health professionals’ beliefs for unconscious repression and dissociative amnesia, especially amongst eye-movement desensitisation and reprocessing (EMDR) practitioners and other trauma-focussed therapists. EMDR aims to modify current and distant memories, typically for patients diagnosed with posttraumatic stress disorder (PTSD) (De Jongh et al., 2019). Some memory scholars are concerned that EMDR therapy along with contentious memory beliefs held by the therapist (and patient) may unintentionally invoke harmful memory distortion (Kenchel et al., 2022; Otgaar et al., 2021b), a potentially adverse outcome for public health and justice (Otgaar et al., 2022).

A related research gap is understanding UK mental health professionals’ current beliefs about the scientific validity of dissociative identity disorder (DID) – a controversial and severe mental illness. Dissociative amnesia for early childhood trauma is a key diagnostic criterion. Some trauma clinicians argue that enhanced diagnostic training is required for this condition due to current misdiagnosis (Brand, 2016; Loewenstein & Brand, 2023; Reinders & Veltman, 2021). Other scholars contend that the diagnosis is scientifically fraught and not necessarily trauma-related (Dodier et al., 2022; Luauté & Pope, 2024). Uncritical belief in dissociative amnesia may cause some trauma-focussed therapists to search for presumed dissociated or repressed memories of childhood trauma and overlook other internal or external factors that may better explain current distress (Fahy, 1988; Paris, 2023; Yapko, 1994b).

Another research gap concerns professionals’ beliefs about the underlying causes of a more prevalent dissociative disorder, psychogenic non-epileptic seizures (PNES), also termed dissociative seizures (Hingray et al., 2022; Reuber, 2019) and formerly called medically unexplained symptoms (Kemp et al., 2013). Whilst childhood sexual trauma is a diagnostic risk factor, it is not a necessary criterion (*Diagnostic and Statistical Manual of Mental Disorders Fifth Edition, Text Revision* (DSM-5-TR; American Psychiatric Association, 2022, p. 362; Yang et al., 2023)). Recent research suggests that emotional neglect (Ludwig et al., 2018) and socioeconomic deprivation (Goldstein et al., 2019) are often key factors and that prior trauma may be absent. Therapists’ erroneous belief in repression or dissociative amnesia and prior assumptions about the aetiology of this condition may lead to the use of problematic techniques to aid therapeutic memory retrieval.

The current study provides novel data on UK mental health professionals’ beliefs about repression and dissociative amnesia and two dissociative trauma-related disorders, DID and PNES. Focussing on EMDR practitioners’ beliefs and memory recovery practices is especially relevant given EMDR’s global reach, universal accreditation and exclusive focus on memory modification for trauma.

## Why believing in unconscious repression and dissociative amnesia is controversial

*Unconscious repression* is a psychoanalytical idea that memory for traumatic experiences can be involuntarily banished from conscious awareness because it is too painful to consciously retain (for its Freudian roots see, Crews, 1995; Ellenberger, 1970; McNally, 2003, pp. 159–168). The notion flourished in the United States in the 1980s–1990s. Proponents of repression theorised that memory for traumatic childhood abuse, e.g., incest, worked differently (e.g., Bass & Davis, 1988; Brown et al., 1998; Fredrickson, 1992; Freyd, 1994 (betrayal theory); Gleaves, 1996; Spiegel, 1997; Terr, 1994; Van der Kolk, 1994 (body memories)). The notion was inextricably linked with the therapeutic belief that such repressed memories could be accurately therapeutically retrieved.

A type of “search and rescue” therapy evolved (Yapko, 1994b, p. 45). Therapists (from varying professional backgrounds) conducted “memory work” (Lindsay & Read, 1995, p. 850) to recover suspected, repressed, trauma memories – despite patients’ absence of recall for trauma. Techniques utilised to recover such memories included drug abreaction, dream analysis, extensive rumination, instructions to remember, feelings work, guided imagery and hypnotic regression. These techniques were criticised by some scholars and clinicians (Lindsay & Read, 1995; Pope & Hudson, 1995). Many of these purported recovered memories were extraordinary e.g., satanic abuse, torture, and murder (see Loftus & Ketcham, 1996; Ofshe & Watters, 1996; Pendergrast, 1995) and mostly false (see Lanning, 1992; La Fontaine, 1998; Mair, 2013; for retractor studies see Lief & Fetkewicz, 1995; De Rivera, 1997) – the iatrogenic product of risky therapeutic techniques (Lambert & Lilienfeld, 2007; Lilienfeld, 2007).

The notion of repression and associated memory recovery work was discredited during the Memory Wars (Crews, 1995; see Lynn et al., 2023 for a historical overview). The scientific validity of repression was questionable (e.g., Brainerd, 2005; Holmes, 1990; McGaugh, 2003; McHugh, 2008; McNally, 2007; McNally, 2003; Pope & Hudson, 1995; Shobe & Kihlstrom, 1997). Some clinicians at that time also questioned the scientific validity of dissociative amnesia, which closely resembled repression (e.g., Pope et al., 1998). Yet despite concerns, the concept of dissociative amnesia gained (and has retained) official clinical recognition (*Diagnostic and Statistical Manual of Mental Disorders Fourth edition*(DSM-IV); American Psychiatric Association, 1994; *Diagnostic and Statistical Manual of Mental Disorders Fifth Edition, Text Revision* (DSM-5-TR); American Psychiatric Association, 2022 (p.337), see Staniloiu & Markowitsch, 2014 for a brief historical overview)). Some scholars argue that dissociative amnesia is indistinguishable from unconscious repression (see Otgaar et al., 2019, Table 2, p. 1080 for a side-by-side comparison). Moreover, some scholars are concerned that the ascendance of dissociative amnesia may herald a reprise of the Memory Wars (Lynn et al., 2023; McNally, 2023; Otgaar et al., 2019).

*Dissociative amnesia* (i.e., retrograde autobiographical amnesia for traumatic or stressful events) is a key idea within the posttraumatic memory model of dissociation (see Brand et al., 2017; Brand et al., 2018; Dalenberg et al., 2012). However, its scientific validity remains contentious (see Mangiulli et al., 2022; Merckelbach & Patihis, 2018; Otgaar et al., 2025b; Patihis, 2023; Pope et al., 1999, 2007, 2023). Advances in neuroscience undermine the existence of any such mechanism (McNally, 2003, pp. 177–181; McGaugh, 2018; Otgaar 2025b; but see Roydeva & Reinders, 2021). Other research shows that children and adults typically remember traumatic experiences (Alexander et al., 2005; Goodman et al., 2003, 2019).

Sceptics of the posttraumatic theory suggest that the sociocognitive model better explains pathological dissociation and associated disorders (see Lilienfeld et al.,^1999^; Lynn et al., 2012, 2014, 2022; Spanos, 1994). They contend more plausible explanations account for claims of dissociative amnesia and repression, e.g., shame, organic amnesia, childhood amnesia, ordinary forgetting or deliberate suppression (Dodier et al., 2024; Mangiulli, et al., 2022; McNally, 2005; but see Staniloiu & Markowitsch, 2018).

Still, recent survey data suggest that therapists’ belief in repression in America and Europe remains “deeply rooted” (Otgaar et al., 2019, p. 1078; Patihis et al., 2014, 2019; see Dodier, 2019 for a bibliometric analysis). Emerging research also indicates that equally strong belief may exist for dissociative amnesia (Mangiulli et al., 2021; Otgaar et al., 2025a; Zappalà et al., 2024). Sceptical scientists are concerned such beliefs may elevate the risk of false memory induction. Understanding mental health professionals’ current memory beliefs remains an important research issue.

## Mental health professionals’ past and current beliefs and modern memory recovery practices

During the 1990s, survey data showed that belief in unconscious repression was prevalent, and memory recovery practices were not rare (see Andrews et al., 1995, UK-chartered psychologists; Poole et al., 1995, UK psychotherapists; Yapko, 1994b, US psychotherapists). Later research showed that contentious memory beliefs were held by Canadian social workers, psychologists and psychiatrists (Legault & Laurence, 2007) although psychiatrists were more sceptical. In Norway, Magnussen & Melinder (2012) found that licenced psychologists’ understanding of memory function was no better than the lay public. In a later US survey, Patihis et al. (2014) found that internal family systems therapists and neurolinguistic programmers (non-mainstream therapies) showed increased belief in repression compared with clinical psychologists. Another study (Otgaar et al., 2019) reviewed data from 21 international surveys. Their study data suggests that clinical psychologists’ beliefs in repression have increased. Otgaar et al. (2019) found that 76% of surveyed clinical psychologists (*n* = 1586) believed in repression in surveys conducted since 2010, compared with 61% (*n* = 719) during the 1990s. A related research concern is understanding the prevalence and therapeutic context of new (purported) memory recovery.

Patihis and Pendergrast (2018) investigated the prevalence of new therapeutic memory recovery (for childhood abuse) by therapy type, amongst the US lay public (N = 2,326). They found that 5% of the total sample (4% weighted) recovered purported memories of childhood abuse (emotional, physical and sexual) of which they were previously unaware. Moreover, new memory recovery was twenty times more likely when therapists discussed the possibility of repressed memories. Some therapies were associated with higher rates of new memory recovery, e.g., attachment therapy, 57% (*n* = 8) and internal family systems therapy, 23% (*n* = 10). Salient to this study, they also found that memory recovery during EMDR – now a mainstream trauma treatment for PTSD – was a non-trivial 13% (*n* = 23). In addition, 13% (*n* = 16) of those participants who reported recovered memories also came to believe they suffered from DID. Notably, their data suggests that DID diagnoses (for which dissociative amnesia is a key component) may be increasing. Between 2015 and 2017, 30% of study participants who experienced new memory recovery came to believe they had DID, compared with 18% and 12% between 1990 and 2014. However, the objective accuracy of such memory recovery has not been verified. (See also Dodier et al., 2019 for recovered memory reports in France).

Relatedly, Zappalà et al. (2024) analysed Italian cognitive behavioural therapists and trainees’ beliefs, and practices associated with memory recovery. They found 78% (*n* = 315) agreed or strongly agreed that “traumatic memories are often not accessible to consciousness” and 83% (*n* = 333) strongly agreed or agreed that “the mind is able to unconsciously block the memories of traumatic experiences” (see Table 1, p. 28). Moreover, 50% (*n* = 98) of their participants sometimes told patients of the possible existence of memories for trauma for which patients were unaware, and 42% (*n* = 82) always discussed that past traumatic experiences may be the root of current psychological symptoms.

**Table 1.** Study group frequencies for biological sex, age, practice type and experience.

Participant group	N	Biological sex		Age	Practice type		Clinical Experience
		Female *n* (%)	Male *n* (%)	M (SD)	NHS *n* (%)	Private *n* (%)	Years M (SD)
Lay public	419	213 (50.8)	206 (49.2)	40.1 (14.2)	n/a	n/a	n/a
Non-trauma-focused	92	66 (71.7)	26 (28.3)	54.8 (10.3)	35 (38.0)	66 (71.7)	19.1 (11.9)
Trauma-focused EMDR	62	54 (87.1)	8 (12.9)	53.7 (11.0)	24 (38.7)	51 (82.3)	20.2 (10.0)
Trauma-focused (Other)	24	17 (70.8)	7 (29.2)	54.2 (9.34)	2 (8.3)	24 (100.0)	12.7 (10.1)

Note: n/a means “not applicable”.

Zappalà et al. (2024) also found that 29% (*n* = 116) of participants strongly agreed and 64% agreed (*n* = 256) that “memories of traumatic experiences can be recovered through psychotherapy”. Relatedly – and significant to the instant study – a large majority of participants, 79% (*n* = 316) said that EMDR was useful for recovering memories, and 14% (*n* = 57) believed that EMDR was the only therapeutic choice. Notably, Zappalà et al. (2024) found significant correlations between patients who recovered new traumatic memories and therapist-patient discussion on the “existence of memories of traumatic events of which they may be unaware” (item 11), *r*(196) = 0.392, *p* < 0.001 and therapist discussion of the “importance of traumatic events in the genesis of their disease”, *r*(196) = 0.284, *p* < 0.001.

Schemmel et al. (2024) also surveyed memory assumptions, memory recovery and therapeutic recovery techniques amongst German psychotherapists (N = 258). Their data showed that 78% (*n* = 201) of participants reported one or more patients (a median of 20%) recovered new memories in therapy. Significant to the instant study, Schemmel et al. (2024) also found that the diagnosis of posttraumatic stress disorder (PTSD) was most frequently associated with purported new memory recovery (32%, *n* = 64). Also, 83% (*n* = 193) of answering participants reported that their patients assumed an association between unrecalled prior traumatic experience and current symptoms.

The most common memory recovery techniques used were the hypnoanalytic affect bridge, used by 48% of participants (*n* = 44), dream interpretation 38% (*n* = 34), EMDR, 23% (*n* = 21) and imaginative psychotherapy, 19% (*n* = 17). Only a minority (a median of 5%) of participants doubted the authenticity of recovered such memories. Schemmel et al. (2024) concluded that the risk of false memory induction via suggestive techniques, e.g., mental imagery, remains a relevant research issue. These suggestive memory recovery techniques were recognised as potentially harmful during the Memory Wars (Lindsay & Read, 1995) and highlighted as “problematic” by Lynn et al. (2015) p. 212. Lynn et al. (2015) compiled an eleven-point “Hypothesized path of false memory creation” (p. 233); heading their list were “Cultural beliefs that link the idea of abuse with psychopathology and the repression of traumatic memories” (p. 233). Notably, their list also included therapist support for these beliefs and therapeutic practices that aimed to unlock amnesia for past trauma.

Most recently, an Irish survey found a high degree of belief in unconscious repression amongst the lay public and talking therapists (Murphy et al., 2025). They also found that memory recovery during therapy was not rare (8%, *n* = 26), echoing the findings of Patihis et al. (2014). Also, 41% (*n* = 15) of talking therapists reported recovering memories during therapy (i.e., clients had no prior knowledge of the memory prior to therapy). Their data also indicated that professional education on scientific memory research was insufficient.

Overall, mounting evidence indicates that new traumatic memory recovery during EMDR treatment is not rare; however, the authenticity of such memory recovery is unknown. Understanding the entire therapeutic journey of memory recovery during EMDR and the context in which it evolves is important. This journey includes examining variables that may positively or negatively impact on memory recovery *prior to* the bilateral treatment phase, e.g., therapist and patient beliefs about memory for trauma, and EMDR techniques used to locate target memories for treatment.

Beneficial memory modification is the main therapeutic aim of EMDR. Arguably, whilst only a small percentage of such newly recovered memories may be objectively inaccurate or false, they potentially constitute non-beneficial, harmful clinical outcomes (e.g., Lambert & Lilienfeld, 2007). Arguably, even a low rate of non-beneficial outcomes is non-trivial in health and justice contexts and warrants professional regulators and trainers’ attention (see Wade et al., 2025 for an analogous argument on false memory induction and the importance of small percentages). Whether the convergence of the EMDR theory, erroneous memory beliefs and problematic memory recovery practices might elevate that the potential for false memory development is therefore an important research area (Kenchel et al., 2022; Otgaar et al., 2021b).

## May EMDR practitioners’ memory beliefs, theory and practices pose an increased risk of harmful (false) memory recovery?

EMDR is a widely recommended, integrative, memory treatment for PTSD and stress-related conditions (De Jongh et al., 2019; De Jongh et al., 2022; NICE, 2005, 2018; WHO, 2013, 2018) and diverse mental health problems (Shapiro, 2014; but see Cuijpers et al., (2020a, 2020b; Phillips et al., 2022). EMDR works by dampening the vividness and emotionality of existing trauma memories (Shapiro, 2001, 2018) using bilateral stimulation via eye movements, binaural tones or tapping. Simultaneously, the patient envisages or thinks about the existing traumatic memory. Many scientists have debated the scientific validity and testability of EMDR theory (e.g., Lohr et al., 2015; Muris & Merckelbach, 1999; Ost & Easton, 2006). More recently, Rosen (2023) has questioned the accuracy of Shapiro’s “origin story” (p. 289) – her discovery of an eye-movement effect during a walk in the park (Shapiro, 1989a). Rosen (2023) presents evidence that ideas drawn from neurolinguistic programming may have shaped Shapiro’s theory. However, we skirt the underlying scientific controversy. Instead, this study focuses on a growing research concern namely, EMDR’s potential for eliciting non-beneficial side-effects, especially susceptibility to memory recovery for non-experienced events.

Recent research findings on EMDR and its potential for negative memory modification are mixed (see Kenchel et al., 2022; Otgaar et al., 2021b for reviews). In their experiment, Houben et al. (2018) found that although participants’ memory vividness and emotionality were reduced after the eye-movement phase, there was some evidence of increased memory inaccuracy and a misinformation effect. However, follow-up studies did not replicate their findings (Kenchel et al., 2022; Van Schie & Leer, 2019). Yet eye movements may increase spontaneous false memories and reduce memory accuracy after a delay (Houben et al., 2020; Leer & Engelhard, 2020).** Interestingly, Littel et al. (2017) found no evidence of an expectancy effect when study participants received priming information on how EMDR worked, prior to receiving eye movements. Researchers are now investigating whether EMDR practitioners’ memory beliefs and erroneous therapeutic guidance may be associated with false memory recovery during therapy.

Wessel (2018) surveyed Dutch EMDR practitioners and found that 93% (*n* = 457) agreed traumatic memories could be blocked. Similarly, Houben et al. (2021b) also found high agreement amongst Dutch EMDR practitioners, for the unconscious “blocking out” of traumatic events (Study 1: 92%), percentage agreement (*n* = 11); Study 2: 71% (*n* = 29). Also, 75% (*n* = 9) viewed memory recovery during EMDR as most likely authentic (in a fictious case vignette). Interestingly, these study participants showed adequate scientific understanding for other aspects of memory function, e.g., mostly disagreeing, that memory cannot be influenced by suggestion and that memory of everything is stored permanently in the brain and agreeing that very vivid memories can be false, and that memory is reconstructive.

In another study, Houben et al. (2021a) examined the potential adverse impact of erroneous scientific guidance in the Dutch EMDR protocol, i.e., that memory works like a video recorder and is accurately stored like a photograph. Researchers concluded that the converging effect of eye movements, therapists’ beliefs, and erroneous memory instructions may encourage an expectancy effect to recall an emotionally vivid memory (see also, Jones & McNally, 2022; Macrae et al., 2002 on expectancy effects).

Reviewing this growing EMDR research strand, Otgaar et al. (2021b) considered EMDR therapists’ beliefs for unconscious repression and authentic memory retrieval may be “potentially perilous” and encourage EMDR practitioners to “suggestively search for hidden memories” and encourage “the creation of false memories” (p. 1258) a potentially adverse therapeutic outcome. Bolstering this concern is recent evidence of such false memory creation in an Italian court case (Otgaar et al., 2022). The EMDR psychotherapist (a doctor) was found guilty of causing serious (unintentional) psychological injury by facilitating false memories of non-experienced sexual abuse. Treatment sessions were recorded which enabled memory experts to identify the controversial, suggestive techniques that invoked the false memories. Serious (mistaken) criminal allegations may occur in the context of EMDR that pose a real threat to justice (Otgaar et al., 2021b). Yet therapeutic sessions are usually confidential and unrecorded. Thus, controversial beliefs and treatment techniques based on flimsy or scientifically contentious theories will typically be difficult to prove. Arguably, EMDR’s underlying Adaptive Information Processing theory (AIP; Royal College of Psychiatrists, 2021b; Shapiro, 2014, 2018) may reinforce contentious memory beliefs and risky memory recovery. However, this model has received scant scientific attention.

The AIP model (Shapiro, 2010, 2014, 2018) underpins EMDR. Lohr et al. (2015) argued that the AIP model has “little scientific basis” (p.284) and Hase et al. (2017) note the neurobiological basis is unclear. Its key idea is that past, unprocessed (implicit) memories “are the cause of a number of mental disorders” including psychosomatic disorders (Hase et al., 2017, p. 2). It posits that trauma memories are sometimes dysfunctionally stored in the brain (out of conscious awareness). Such unprocessed memories can manifest themselves as distressing physical symptoms, feelings or thoughts (De Jongh et al., 2010, 2022). Thus, the patient may be unaware of the hidden, unprocessed negative memory when they present in therapy. Patients receive a psychoeducation phase that explains this AIP memory model. Knowledge of the AIP model is required knowledge for UK EMDR NHS practitioners (Royal College of Psychiatrists, 2021b). Potentially, this model may reinforce practitioners’ (and clients’) prior belief in repression or dissociative amnesia (controversial notions) and fuel a mutual expectation, that hidden memories await discovery.

A related aspect of interest to memory scholars (that seems to have received limited scientific attention) is the “floatback” technique (Browning, 1999; De Jongh et al., 2010; Shapiro, 2001). Some readers may presume that clients/patients attending EMDR always know the source of their distress and trauma memory. For sure, this may be the case with patients who have been diagnosed with PTSD and suffer nightmares attributable to a known motor vehicle accident. However, EMDR theory implies this is not always the case and that the current presenting problem may not represent the source of the current complaint. The floatback technique can be used to locate the relevant target memory or “touchstone event” (De Jongh et al., 2010, p. 13) “stored outside conscious awareness” (Paterson, 2020). It is recommended in the UK for patients diagnosed with Complex PTSD (Royal College of Psychiatrists, 2021c).

Described as a form of free association (De Jongh et al., 2010), patients visualise or recall the problematic negative event and then are told “ … just let your mind float back to an earlier time in your life – don’t search for anything – just let your mind float back and tell me the first scene that comes to mind where you had something similar … ” (De Jongh et al., 2010, p. 13). There is a step-by-step script for this procedure. This type of imaginative procedure to elicit forgotten/implicit memories along with the therapist and/or client's belief in repression or dissociative amnesia is potentially problematic (Lynn et al., 2015). New purported memory recovery is a known feature of EMDR (Shapiro, 1989a, 1989b, 2001, 2018) and may be accompanied by increased distress, a known side-effect of EMDR (NHS, 2023) or early treatment dropout (see Lewis et al., 2020). However, these new memories may be accurate or partly or wholly false.

### EMDR and the treatment of complex dissociative disorders

The disorders of DID (formerly, multiple personality disorder) and PNES (psychogenic non-epileptic seizures) are considered trauma-related and potentially life-threatening conditions. For diagnostic and associated features and risk factors of DID, see DSM-5-TR pp. 330–334, and for PNES, see DSM-5-TR pp. 360–362. However, some patients report no history of trauma (e.g., for DID, see DSM-5-TR, pp. 331–332; for PNES, see Kanemoto et al., 2017). Such patients typically have high levels of self-harm and comorbidity (for DID, see DSM-5-TR, p. 334; for PNES, DSM-5-TR, p. 363). Arguably, mental health professionals who believe in repression or dissociative amnesia (especially for childhood trauma) may pose an elevated health risk to such patients. For example, EMDR or other therapeutic practitioners may attempt to recover suspected dissociated or repressed trauma memories to explain and alleviate current symptoms. However, such attempts may cause increased psychological distress and even invoke false memories for non-experienced events.

DID has long been a controversial condition (Aldridge-Morris, 1989; Fahy, 1988; Ganaway, 1989; Spanos, 1994); and its aetiology remains contentious (e.g., Luauté & Pope, 2024). No biological markers for the disorder exist. Posttraumatic scholars claim that the disorder is caused by extreme childhood abuse. Dissociative amnesia is said to protect such victims by rendering the main personality amnesic for the memory of the trauma (Brand et al., 2014, 2018; Loewenstein, 2018).

However, sociocognitive scholars question the scientific validity of this hypothesis and the correlational strength between trauma and dissociation (Patihis & Lynn, 2017; Lynn et al., 2014). Moreover, dissociation can occur in the absence of trauma (Briere & Runtz, 2015). Sociocognitive scholars suggest that multiple internal and external variables better explain the condition, e.g., (internal factors) sleep dysfunction, elevated absorption, creative imagining and fantasy proneness (see Lilienfeld & Lynn, 2015; Lohr et al., 2015; Lynn et al., 2012, 2014, 2015, 2019; Merckelbach et al., 2022) and (external factors) social contagion (Luauté & Pope, 2024) and media influences (North et al., 1993; Spanos, 1994).

PNES (also called dissociative seizures, Reuber, 2019) are prevalent across all cultures, but their aetiology is poorly understood (Huff & Murr, 2024). These patients experience non-epileptic seizures and random bodily symptoms of psychological origin, but a history of trauma “is not a necessary or sufficient factor” (Jones & Rickards, 2021, p. 202). International consensus ranks PNES among the top three neuropsychiatric problems (Kanemoto et al., 2017). The condition poses considerable diagnostic and treatment challenges (see Vijay & Reuber, 2024). Mindfulness or body-focussed therapy is recommended (Vijay & Reuber, 2024) but no UK treatment guidelines exist (Kanemoto et al., 2017). Kanemoto et al. (2017) observed that in the UK, “psychiatrists, psychologists, and psychotherapists not working in epilepsy centers have a very limited understanding of PNES” (p. 313). If these patients are mishandled or misunderstood, their recovery may be compromised (Reuber, 2019). Some clinicians highlight childhood sexual abuse as a potential risk factor (Alper et al., 1993; Jones & Rickards, 2021; Karatzias et al., 2017; Yang et al., 2023). Other researchers argued that no single explanatory factor explains the disorder (Kanemoto et al., 2017), and childhood sexual abuse does not pose an elevated risk (Goldstein et al., 2021). In short, the aetiology of PNES is uncertain (see Hingray et al., 2022).

## UK research on mental health professionals’ beliefs

Between 2007 and 2009, Ost et al. (2013, 2017) surveyed the memory beliefs of UK chartered clinical psychologists and hypnotherapists including belief in repression. They found that 70% (*n* = 87) of psychologists and 89% of hypnotherapists (*n* = 78) strongly agreed that “The mind is capable of unconsciously ‘blocking out’ memories of traumatic events”, a concept akin to unconscious repression (using a four-point Likert-scale). Overall, they found that psychologist participants’ beliefs were more in line with scientific consensus than hypnotherapists. Interestingly, their data showed that more clinical psychologists had seen a case of DID/MPD than hypnotherapists; 43.7% of clinical psychologists had seen a case (range = 1–50, M = 5.03) compared with 33.7% of hypnotherapists (range = 1–30, M = 3.96) (Ost et al., 2013). The other UK study of relevance is Kemp et al. (2013).

Kemp et al. (2013) surveyed the beliefs of trainee psychologists, neurologists and psychiatrists about the aetiology of medically unexplained symptoms (MUS) (later renamed PNES). They found all three groups held memory beliefs that were scientifically contentious. Only a small proportion of psychologists and psychiatrists doubted the reality of “massive repression” and an overwhelming majority believed that childhood sexual abuse was a medium to high-risk factor for developing MUS/PNES. To our knowledge, no further research exists on UK mental health professionals’ memory beliefs for trauma.

## The current study

The present study fills an important research gap by collecting new survey data on UK mental health professionals’ beliefs for traumatic memory function, especially unconscious repression and dissociative amnesia. Both these notions are scientifically contentious. In the past, such beliefs were associated with adverse therapeutic outcomes, e.g., iatrogenic memories for non-experienced trauma (Loftus, 1993; Lynn et al., 2023; McNally, 2023) and increased psychological harm (Lambert & Lilienfeld, 2007). We also surveyed the lay public for comparison purposes.

As summarised above, belief in repression remains high amongst mental health professionals in the United States (Patihis et al., 2014) and in Europe (Mangiulli, et al., 2022; Murphy et al., 2025; Otgaar et al., 2019; Schemmel et al., 2024; Zappalà et al., 2024). Past UK research data collected between 2007 and 2009 (Ost et al., 2013, 2017) may not reflect current beliefs, and likewise, trainee psychologists’ beliefs were surveyed by Kemp et al. (2013).

Another study aim was to improve memory researchers’ understanding of trauma-focussed therapists’ memory beliefs, whose training may be influenced by the posttraumatic memory model. To our knowledge, no research has compared the beliefs of non-trauma-focussed and trauma-focussed mental health professionals. Trauma-focussed psychological treatments are now abundant (Han et al., 2021; Schnurr, 2017; Wilson et al., 2013). However, the prevalence of trauma-focussed and trauma-informed practitioners in the UK, their beliefs about trauma, professional backgrounds and practice type are all unknown. Any mental health professional can use the title of “trauma-focussed” or “trauma-informed” therapist in the UK. Even the titles “therapist” and “psychologist” are unprotected. This study aimed to fill an important knowledge gap by surveying and comparing beliefs held by both non-trauma and trauma-focussed UK mental health professionals.

Another key study aim was to investigate UK EMDR practitioners’ memory beliefs. EMDR is a globally popular, validated trauma treatment. It is of special research interest to memory scientists due to its known memory-changing effects. As shown above, emerging European research suggests that many EMDR practitioners hold contentious memory beliefs. This research also indicates that EMDR is associated with memory recovery (in addition to memory dampening). However, UK EMDR practitioners’ memory beliefs have not yet been surveyed (to the best of our knowledge). To address this gap, we aimed to target EMDR practitioners and capture a representative sample.

Despite the extant international data on mental health professionals’ memory beliefs for repression, research on beliefs about dissociative amnesia – including EMDR practitioners’ beliefs and memory recovery practices – is limited and still emerging. We also considered training differences (for EMDR practitioners) that might exist between the UK and Europe. We therefore concluded that it was premature to formulate firm study hypotheses. For this reason, this study is exploratory only.

Our survey was divided into three sections: demographic information, a memory questionnaire, then questions about dissociative identity disorder, dissociative amnesia and psychogenic non-epileptic seizures. In our memory questionnaire, we asked nine questions, eight items reflected statements from prior research (Kemp et al., 2013; Patihis et al., 2014; Yapko, 1994a).

Our main research questions were: (i) What proportion of UK mental health professionals believe in repression and dissociative amnesia, (ii) Are the memory beliefs of non-trauma-focussed therapists, trauma-focussed EMDR practitioners and other trauma-focussed therapists similar? and (iii) What proportion of mental health professionals believe in the possibility of false memories? Other research aims were to investigate beliefs about DID and the underlying causes of PNES.

## Method

### Participants

A total of 597 UK citizens completed the study survey: lay public (*n* = 419) and mental health professionals (*n* = 178). In the total sample (N = 597), participants’ ages ranged from 18 to 77 (M_age_ = 44.4; SD = 14.7). Regarding biological sex, 58.6% (350) were female, and 41.4% (247) were male. Overall ethnicity for survey participants was 89.9% white and 4.4% Asian/Asian British. All participants gave consent for their answers to be used for scientific purposes. See Supplemental Materials Table S1 for further detail on lay public and mental health professional participants’ ethnicity.

The current survey and procedures were part of a larger study approved by the Science and Health Faculty Ethics Committee of the University of Portsmouth (SHFEC 2023–113) in which legal professionals’ memory beliefs were also examined (see Radcliffe & Patihis, 2024). Mental health professional participants received no financial or other incentive to enter the survey.

#### Lay public

Any British citizen aged 18 years or over was eligible to enter the survey. Lay participants were recruited via the marketing company Prolific (https://www.prolific.co/) and received £1.64 for taking part. In total, 451 persons attempted the survey’ however, 31 cases were removed where the survey completion time was under 90 s. A final total of 419 lay public study participants were retained. Regarding biological sex, 50.8% (213) were female and 49.2% (206) male; M_age_ = 40.1; SD 14.2. Further details about lay participant recruitment, socioeconomic and educational status are found in the Supplemental Materials, p.1.

The survey sample size for the lay public sample was mainly determined by financial constraints. Moreover, in the first study (Radcliffe & Patihis, 2024) – in contrast to the instant study – inferential statistics were conducted; therefore, a G*Power analysis (Erdfelder et al., 1996) was also conducted to guide that study. This analysis suggested a sample size of at least 382 (to detect an effect size of Cohen’s *d* = 0.30, β = 0.90, *p* = .05).

#### Mental health professionals

Regarding our approach to study sample size, we aimed to recruit as many mental health professionals from as many different domains as possible, with no limit on sample size. A total of 178 mental health professionals completed the study survey. Their ages ranged from 26 to 76 (M_age_ = 54.3; SD 10.3). Regarding biological sex, 77% (137) were female and 23.0% (41) male. Two participants identified as having a different birth gender. One participant identified as non-binary and one wrote, “Don’t believe in gender identity”.

Any qualified mental health or recognised therapeutic professional practising in the UK (registered or unregistered) was eligible to enter the study. Participant recruitment occurred in two tranches during 2023 and 2024. All mental health professional participants were self-selecting and mainly entered the survey by responding to in-house advertisements (only accessed by a specific professional group) or generic email invitations to join the study – the e-address was sourced from the organisation’s website. We also increased EMDR practitioner participants – our study group of key interest. We conducted a second recruitment phase in February 2024 via an advertisement in an in-house EMDR journal. Our Internal Family Systems participants were recruited via a generic email to their public online membership directory located on the IFS website https://www.info@ifstuk. For further detail on mental health professionals’ recruitment, data collection and professional practice details, see Supplemental Materials, Table S2, p. 2.

Table 1 shows demographic and practice information for lay public and mental health professional participants. The study aim was to compare beliefs between mental health professionals whose clinical orientation was or was not trauma-focussed. To achieve this, mental health professional participants were assigned to three groups according to their therapeutic orientation as shown in Table 1, in the left-hand column. The non-trauma-focussed group, (*n* = 92) comprised all mental health professional study participants *except* participants who ticked one or more of the following professional titles: EMDR therapist/practitioner, internal family systems therapist, trauma-informed therapist, and/or dissociative disorder specialist. EMDR is a trauma-focussed treatment. Due to the special study focus on EMDR, participants who identified as EMDR therapists/practitioners were analysed as a discrete group, which we termed “Trauma-focussed EMDR” (*n* = 62). The “Trauma-focussed (Other) group” (*n* = 24) comprised all study participants excluded from the non-trauma-focussed group, *except* EMDR practitioners. We highlight that due to our targeted recruitment process, our overall study sample is unrepresentative of the diversity of mental health professionals, therapy type and psychoanalytical orientation within the UK.

We also sought to measure the distribution of therapists practising in the private (fee paying) and/or public (no fee), National Health Service (NHS) sectors. In the UK, a diversity of talking therapists exist. In the private health sector, multiple professional bodies regulate mental health professionals from differing theoretical and educational backgrounds (see Supplemental Materials Table S2 for examples of these organisations). Regulatory and quality standards are not centralised. Non-mainstream therapists with more scientifically controversial theories, e.g., neurolinguistic programming, may be more plentiful in the private sector compared with the public NHS sector. Moreover, in the private sector, there is no restriction on whom EMDR practitioners may treat. Whereas, within the NHS, EMDR practitioners may only treat patients with PTSD or Complex PTSD. For this reason, understanding practice type alongside individual beliefs was relevant data. Table 1 shows mental health practitioners’ practice type, i.e., UK National Health Service (NHS) and/or private sector.

Mental health professional participants by nation: 5.1% (*n* = 9) practised in Scotland, 1.1% (*n* = 2) in Wales, 2.2% (*n* = 4) in Northern Ireland and 89.9% (*n* = 160) in England. Thirty-seven participants (20.8%) also practised online. Overall, 34.3% (*n* = 61) of mental health professional participants practised within the National Health System and 79.2% (*n* = 141) in the private sector. Some practitioners practised in both sectors.

Mental health professional participants selected their therapeutic title(s) from a drop-down list. A text box allowed them to write their professional title if it did not appear on the list. In the UK, individual mental health practitioners may use multiple therapeutic methods and professional titles. For this reason, participants were permitted to tick more than one professional descriptor. “EMDR practitioner” (*n* = 62) was the most frequently selected title, comprising 34.8% of all mental health professional participants, followed by “psychotherapist” 30.3% (*n* = 54), “trauma-informed therapist”, 22.5% (*n* = 40), “counsellor” 21.3% (*n* = 38) and “hypnotherapist” 19.1% (*n* = 34).

The most frequently selected professional titles in each study group were: in the non-trauma-focussed group, hypnotherapist, 27.2% (*n* = 25), clinical psychologist, 26.1% (*n* = 24),and then psychotherapist, 23.9% (*n* = 22); in the trauma-focussed EMDR group, “trauma-informed therapist” 43.5% (*n* = 27), “psychotherapist” 35.5% (*n* = 22), “counsellor” 32.3% (*n* = 20) and “cognitive behavioural therapist” 30.6% (*n* = 19); and in the trauma-focussed (Other) group, internal family systems therapists, 62.5% (*n* = 15), trauma-informed therapist, 54.2% (*n* = 13) and psychotherapist 41.7% (*n* = 10). For complete distribution and percentages of all professional titles and descriptive details for all three study groups, see Supplemental Materials Table S4, p. 6–7.

Ten study participants (all located in the non-trauma-focussed group) self-identified as expert witnesses. Their titles were: Human Givens therapist (*n* = 1), Psychiatrist (*n* = 4), Clinical Psychologist (*n* = 4) and Psychotherapist (*n* = 1). See Supplemental materials, p. 5 for further demographic and professional detail.

### Materials and procedure

This anonymous online study used the Qualtrics software platform (www.qualtrics.com) to create and host the survey. Participants completed the survey at a remote location of their choice in their own time, on any digital online device. It took between 10 and 15 minutes to complete. On opening the survey, prospective participants read a Participant Information Sheet. This explained that the main study aim was to investigate what individuals believe about memory function; no reference was made to repression, trauma, or dissociative amnesia. They were told that they could skip any question or leave the study at any time. After giving informed consent, Section 1 asked all participants general demographic questions, i.e., age, gender, nationality and ethnicity. Lay public participants were also asked about educational attainment (for details see Radcliffe & Patihis, 2024; Supplemental Materials). Mental health professional participants were asked about professional title, clinical experience, practice type and professional affiliation.

Next, all participants saw an identical Section 2, the memory belief questionnaire (MBQ). This consisted of nine statements about memory function and a tenth question about participants’ source(s) of knowledge. Survey item wording and Likert-rating scales for items 1–8 were drawn from prior studies, as shown in Tables 2–4. Item 9, “*Dissociative amnesia prevents a person from recalling traumatic experiences*” was novel (see Supplemental Materials, Table S5 for all survey statements and Likert scales). The internal consistency across all nine statements was Cronbach’s alpha = .67.

**Table 2.** Percentage agreement for MBQ items 1 & 2 within study groups.

Item number and statement	% Agreed to some degree; (*n*)				
	Lay public N = 419	Non-trauma-focused *n* = 92	Trauma-focused EMDR *n* = 62	Trauma-focused (Other) *n* = 24	Therapists (1994) *n* = 864
(1) The mind is like a computer, accurately recording events as they actually occurred	45.2 (189)	15.2 (14)	17.7 (11)	8.3 (2)	33.1 (286)
(2) I believe that early memories, even from the first year of life, are accurately stored and retrievable	28.6 (120)	22.8 (21)	38.7 (24)	37.5 (9)	40.5 (350)

Note: These two statements are copied from Yapko (1994a, 1994b). The fully anchored 4-point Likert was: *disagree strongly, disagree slightly, agree slightly, agree strongly*. “% Agreed to some degree” = the total percentage of responses to *agree strongly* and *agree slightly*. The study groups were: the non-trauma-focussed group, comprising all mental health professional participants *except* participants who ticked the professional title(s): eye-movement desensitisation and reprocessing (EMDR) practitioner, Internal Family Systems therapist, trauma-informed therapist, and dissociative disorder specialist; the trauma-focussed EMDR group and the trauma-focussed (Other) group comprising participants excluded from the non-trauma-focussed group except for EMDR practitioners.

**Table 3.** Percentage agreement for MBQ items 3 through 7 within participant groups.

Item number and statement	% Agreed to some degree; (*n*)				
	Lay public N = 419	Non – trauma-focused *n* = 92	Trauma-focused EMDR *n* = 62	Trauma-focused (Other) *n* = 24	Clin. Psy (2014) *n* = 58
(3) Memory is constantly being reconstructed and changed every time we remember something	89.7 (376)	93.5 (86)	96.8 (60)	95.8 (23)	98.3 (57)
(4) The memory of everything we have experienced is stored permanently in our brains even if we cannot access all of it	57.1 (239)	53.2 (49)	50.0 (31)	54.1 (13)	44.8 (29)
(5) Traumatic memories are often repressed (which means the person cannot remember the traumatic event due to a defence against painful content).	90.4 (377)	78.1 (71)^a^	82.3 (51)	91.7 (22)	60.3 (35)
(6) Repressed memories can be retrieved in therapy accurately	75.4 (316)	57.2 (52)^b^	56.5 (35)	75.1 (18)	43.1 (25)
(7) Hypnosis can accurately retrieve memories that previously were not known to the person	60.9 (255)	52.2 (46)^c^	38.7 (24)	55.0 (11)^d^	36.2 (21)

Note. Items 3–7 are copied from Patihis et al., 2014. The far-right column shows clinical psychologist practitioners’ responses (Patihis et al., *n* = 58). Items 3–7 had a fully anchored 6-point Likert-scale: *strongly disagree, disagree, slightly disagree, slightly agree, agree, strongly agree*. “% Agreed to some degree” are the percentage responses to: “*slightly agree, agree, or strongly agree*.” Participant groups from left to right: the lay public; the non-trauma-focussed group – all mental health participants *except* eye-movement desensitisation and reprocessing (EMDR) practitioners, Internal Family Systems therapists, trauma-informed therapists, and dissociative disorder specialists; the trauma focussed EMDR group, EMDR practitioners only and the trauma-focussed (Other) group – study participants excluded from the non-trauma-focussed group, *except* EMDR practitioners. Due to missing cases: ^a^ *n* = 91; ^b^ *n* = 91; ^c^ *n* = 88; ^d^ *n* = 20. In the lay public sample, N = 417 for item 5, due to missing cases.

**Table 4.** Percentage agreement for MBQ items 8–9 within participant groups.

Item statement	% Agreed to some degree; (*n*)				
	Lay public *N* = 419	Non- trauma-focused *n* = 90^a^	Trauma-focused EMDR *n* = 62	Trauma-focused (Other) *n* = 24	Clin. Psy. (2013) N = 375
(8) It is possible to develop false memories for abuse/trauma that did not happen	63.6 (266)	71.1 (64)	66.1 (41)	60.8 (14)^b^	84.9 (318)
(9) Dissociative amnesia prevents a person from recalling traumatic experiences	91.1 (381)	84.4 (76)	90.3 (56)	95.8 (23)	n/a

Note*.* Item 8 statement wording and responses replicate Kemp et al. (2013). The far-right column shows trainee clinical psychologists’ responses (Kemp et al., *n* = 375) for comparison. Item 8 had a 5-point Likert-scale: “*disagree, somewhat disagree, don’t know, somewhat agree, agree.* “Agreed to some degree” for item 8 = the total percentage of responses to: *somewhat agree* and *agree*. Item 9 was new. Item 9 had a fully anchored 6-point Likert-scale: *strongly disagree, disagree, slightly disagree, slightly agree, agree, strongly agree*. “% Agreed to some degree” = the total percentage of responses for: *slightly agree, agree, or strongly agree*.” ^a^ Due to missing cases, *n* = 90. ^b^ Due to one missing case, *n* = 23. Participant groups are as in Tables 2 and 3 above.

MBQ item 10, asked participants to tick the source of knowledge for their answers. Multiple answers were permitted from the following list: professional education, private reading, social media, TV documentaries/films and innate beliefs. A text box option labelled, “*Other*” enabled participants to add another source. After item 10, participants were invited to write a further response: “*Optional response: If the above questions did not allow you to specify your beliefs exactly, please feel free to comment below*.” After completing the MBQ, lay study participants were thanked, debriefed, and guided back to the Prolific platform to receive automatic payment. All mental health professional participants next saw an optional Section 3.

Section 3 replicated four questions from Pope et al. (1999) about DID and dissociative amnesia. Additional questions probed DID prevalence: “*How many cases have you dealt with that met the criteria for a diagnosis of dissociative identity disorder (DID) since 2010?*” and “*How many cases of satanic ritual abuse have you dealt with since 2010*?” Two final questions, (from Kemp et al., 2013), firstly asked participants about the causal relationship between physical symptoms e.g., “non-epileptic seizures” and “blocked out” memories of past trauma and secondly, the degree of risk posed by childhood sexual abuse for developing medically unexplained symptoms. We retained the term “medically unexplained symptoms” even though other descriptors are now used (e.g., PNES or functional dissociative seizures) to preserve study integrity when comparing beliefs between 2013 and 2024. Also, the term MUS is still widely used. Afterwards, mental health professionals were thanked for taking part and debriefed for the lay public.

#### Statistical analysis

All statistical analysis was conducted using IBM SPSS Statistic v27. No inferential statistics are reported due to the small sample sizes.

## Results

In these results, we first show the lay public and mental health professional groups’ responses to the Memory Belief Questionnaire (MBQ) in Tables 2–6. Participants’ sources of knowledge for their MBQ responses are then shown in Table 7. After this, mental health professional participants’ beliefs for dissociative conditions and related issues are shown, e.g., about the scientific status of DID, and the causal relationship between past trauma and dissociative symptoms. Results for the non-trauma-focussed group *include* responses from ten participants who identified as expert witnesses. Experts’ individual responses to items 5, 6, 7, 8, and 9 are briefly discussed below with further detail in the Supplemental Materials, Table S8, p. 14.

**Table 5.** Group percentages for all Likert-scale responses for MBQ item 5.

	Practice type %	Traumatic memories are often repressed (which means the person cannot remember the traumatic event due to a defence against painful content)						
		Likert response%						
Participant group	NHS	Private	Strongly disagree	Disagree	Slightly disagree	Slightly agree	Agree	Strongly agree
Non-trauma-focused								
Hypnotherapists	12.0	96.0	4.00 (1)	0.00 (0)	12.0 (3)	12.0 (3)	56.0 (14)	16.0 (4)
Clinical psychologists	66.7	50.0	8.30 (2)	20.8 (5)	12.5 (3)	33.3 (8)	25.0 (6)	0.00 (0)
Trauma-focused								
Internal family systems	0.00	100.0	0.00 (0)	0.0 (0)	13.3 (2)	6.70 (1)	40.0 (6)	40.0 (6)
EMDR therapists	38.7	82.3	1.60 (1)	8.10 (5)	8.10 (5)	33.9 (21)	24.2 (15)	24.2 (15)

Note. Participant numbers for each group: Hypnotherapists, *n* = 25; Clinical psychologists, *n* = 24; Internal family systems, *n* = 15; EMDR therapists, *n* = 62.

**Table 6.** Group percentages for all Likert-scale responses for MBQ item 9.

		Dissociative amnesia prevents a person from recalling traumatic experiences						
Practice type %				Likert response %				
Participant group	NHS	Private	Strongly disagree	Disagree	Slightly disagree	Slightly agree	Agree	Strongly agree
Non-trauma-focused								
Hypnotherapists	12.0	96.0	0.0 (0)	12.0 (3)	4.0 (1)	28.0 (7)	44.0 (11)	12.0 (3)
Clinical psychologists	66.7	50.0	4.2 (1)	4.2 (1)	12.5 (3)	29.2 (7)	50.0 (12)	0.0 (0)
Trauma-focused								
Internal family systems	0.0	100.0	0.0 (0)	0.0 (0)	6.7 (1)	26.7 (4)	46.7 (7)	20.0 (3)
EMDR therapists	38.7	82.3	0.0 (0)	1.6 (1)	8.1 (5)	24.2 (15)	43.5 (27)	22.6 (14)

Note. Participant numbers for each group: Hypnotherapists, *n* = 25; Clinical psychologists, *n* = 24; Internal family systems, *n* = 15; EMDR therapists, *n* = 62.

**Table 7.** Comparing beliefs for the diagnostic inclusion of dissociative identity disorder.

Participant group	Item question: If the DSM-5 were to be revised today, how should it treat the diagnosis of dissociative identity disorder?				
	*n*	Should not be included at all ^a^ % (*n*)	Include only with reservations ^b^ % (*n*)	Include without reservations % (*n*)	No opinion % (*n*)
Non-trauma-focused	76	9.2 (7)	32.9 (25)	22.4 (17)	35.5 (27)
Trauma-focused EMDR	57	3.5 (2)	24.6 (14)	43.9 (25)	28.1 (16)
Trauma-focused (Other)	21	14.3 (3)	47.6 (10)	23.8 (5)	14.3 (3)
American Psychiatrists (1999)	301	15.0 (45)	43.0 (128)	35.0 (106)	7.0 (22)

Note. This table is adapted from “Attitudes Toward DSM-IV Dissociative Disorders Diagnoses Among Board-Certified American Psychiatrists” Pope et al., 1999, *American Journal of Psychiatry*. Their study data – participants were board-certified psychiatrists – is shown for comparison on the bottom line. ^a^The full text of this first response option in the survey was “*Should not be included at all (or included only as an “iatrogenic” phenomenon)*.” ^b^The full text of this response option in the survey was, “*Should be included only with reservations (e.g., only as a proposed ‘diagnosis’)*”. Participant groups are as described in Table 2.

### Percentage agreement analysis

Table 2 compares survey participants’ agreement between the lay public and three mental health professional participants’ groups – non-trauma-focussed, trauma-focussed EMDR and trauma-focussed (Other) – for two items taken from Yapko (1994a). Yapko’s survey results are shown in the column on the far-right. For item 1, “*The mind is like a computer, accurately recording events as they actually occurred*”, lay public participants showed the highest overall agreement, exceeding Yapko’s (1994a) psychotherapists. Participants in the trauma-focussed (Other) group agreed the least with this statement. For item 1, in the trauma-focussed (Other), 8.3% (*n* = 2) chose “Agree slightly” and none chose “Agree strongly”. For item 1, in the Non-trauma-focussed and Trauma-focussed EMDR groups, 14.1% (*n* = 13) and 12.9% (*n* = 8) participants, respectively, chose “Agree slightly” and 1.1% (*n* = 1) and 4.8% (*n* = 3) participants, respectively, chose “Agree strongly”. For item 2, “*I believe early memories, even from the first year of life are accurately stored and retrievable*”, over one-quarter of the lay participants 28.6% (*n* = 120) slightly or strongly agreed with the statement. Amongst mental health professional participants, the non-trauma-focussed group showed the least agreement; 17.4% (*n* = 16) chose “Agree slightly” and 5.4% (*n* = 5) “Agree strongly”. For item 2 in the trauma-focussed EMDR and trauma-focussed (Other) groups, 35.5% (*n* = 22) and 25.0% (*n* = 6), respectively chose “Agree slightly” and 3.2% (*n* = 2) and 12.5% (*n* = 3) and, respectively, chose “Agree strongly”.

Table 3 shows all study groups’ percentage agreement for MBQ items 3–7. These items were copied from Patihis et al. (2014). For item 3, “*Memory is constantly being reconstructed* … ”, all four study groups showed similarly high levels of agreement, exceeding 89%. For item 4, “*The memory of everything we’ve experienced is stored permanently in our brains, even if we can’t access all of it*” more than 50.0% of all study participants agreed to some degree. For item 4, in the trauma-focussed EMDR, 14.5% (*n* = 9) chose “Strongly agree” and 14.5% (*n* = 9) “Agree”. For item 4 in the trauma-focussed (Other) group, 8.3% (*n* = 2) chose “Strongly agree” and 20.8% (*n* = 5) chose “Agree”. For item 5, “*Traumatic memories are often repressed* … ” 75.0% (*n* = 18) of the trauma-focussed (Other) chose “Agree” or “Strongly agree” compared with 53.9% (*n* = 49) of the non-trauma-focussed and 48.4% (*n* = 30) of the trauma-focussed EMDR. For item 6, accurate therapeutic retrieval for repressed memories over 75.0% of the lay public and trauma-focussed (Other) group agreed to some degree. For item 7, “*Hypnosis can accurately retrieve memories* … ”, no participants in the trauma-focussed (other) group selected “Strongly agree”. Whereas, in the non-trauma-focussed group, 4.5% (*n* = 4) of participants chose “Strongly agree” as did 3.2% (*n* = 2) in the trauma-focussed EMDR group. See Table S.6 in the Supplemental Materials for all levels of agreement for the three study groups including the ten expert witness participants, for items 3, 4, 5, 6, 7, & 9.

For item 8, “*It is possible to develop false memories for abuse/trauma that did not happen*”, more than 60.0% of all study participants selected, “Somewhat agree” or “Agree”. Amongst mental health professional participants for item 8, the percentage of “Don’t know” responses and combined, “disagree” or “somewhat disagree” responses were: the trauma-focussed EMDR group, “Don’t know”, 17.7% (*n* = 11); disagreed, 16.2% (*n* = 10); the trauma-focussed (Other) group, “Don’t know”, 13.0% (*n* = 3); disagreed, 26.0% (*n* = 6); the non-trauma-focussed group, “Don’t know”, 16.7% (*n* = 15); disagreed, 12.2% (*n* = 11). For item 9, “*Dissociative amnesia prevents a person from recalling traumatic experiences*”, the highest and strongest level of agreement was seen in the trauma-focussed (Other) group. The total percentage for “Agree” and “Strongly agree” by the trauma-focussed EMDR group was 66.1% (*n* = 41) and the trauma-focussed (Other) was 70.8% (*n* = 17).

To better understand the degree of agreement and disagreement for key items 5 (traumatic repression) and item 9 (dissociative amnesia) within and between individual professional groups, comparisons of all Likert-scale responses were conducted. Responses from the following groups were compared: hypnotherapists (*n* = 25) and clinical psychologists (*n* = 24) (representing 27.2% and 26.1%, respectively, of the non-trauma-focussed group) and internal family systems therapists (IFS) (*n* = 15) (representing 62.5% of the trauma-focussed (Other) group) and trauma-focussed EMDR therapists (*n* = 62).

For item 5, (traumatic repression) IFS therapists and hypnotherapists showed the highest levels of agreement. For item 5, combined percentage scores for “Agree” and “Strongly agree” were IFS, 80% (*n* = 12) and hypnotherapists, 72% (*n* = 18). In contrast, EMDR practitioners’ combined percentage for “Agree” and “Strongly agree” was 48.4% (*n* = 30). Clinical psychologists showed the most disagreement with this item; 41.6% (*n* = 10) disagreed to some degree. No clinical psychologist participants chose “Strongly agree”. Table 5 shows the percentage responses for item 5 (traumatic repression).

Table 6 shows group percentages for all Likert-scale responses for MBQ item 9, dissociative amnesia: “*Dissociative amnesia prevents a person from recalling traumatic experiences*”. Levels of agreement between groups differed from item 5. EMDR practitioners showed increased levels of agreement compared with their responses for repression, item 5. Sixty-six percent of EMDR and IFS practitioners chose “Agree” or “Strongly agree”. IFS therapists had decreased levels of agreement compared with their responses for item 5, but agreement was still high at 66% (*n* = 10), who “Agreed” or “Strongly agreed”. Clinical psychologists also showed increased agreement with dissociative amnesia, although none chose “Strongly agree”.

Finally, the MBQ responses of ten mental health professional participants from the non-trauma-focussed group who self-identified as expert witnesses were analysed. These experts mostly showed beliefs consistent with mainstream science. However, five experts (two of whom were psychiatrists) chose “Agree” or “Strongly agree” item 5, repression. And for dissociative amnesia, 80% (*n* = 8) agreed to some degree. See Table S.8 in the Supplemental Materials for complete responses to MBQ items 5–9 from mental health professional participants who self-identified as expert witnesses.

### Sources of knowledge for memory beliefs

Over 90.2% of mental health professional participants in all three groups selected “*Professional education*” as a source of knowledge. “*Private reading*” was the next most common source, chosen by over 77.4% of participants. “*Innate beliefs*” was selected by 21.7% (*n* = 20) of participants in the non-trauma-focussed group, 19.4% (*n* = 12) of participants in the trauma-focussed EMDR group and 33.3% (*n* = 8) of participants in the trauma-focussed (Other) group.

Lay public participants’ most common source of knowledge was “*Innate beliefs*”, 62.8.0% (*n* = 263), followed by “*Private reading*” 49.2% (*n* = 206) and then “*TV and/or films*”, 46.8% (*n* = 196). See Supplemental Materials Table S7 for all knowledge source frequencies.

An open text box enabled all study participants to add further information about their sources of knowledge. Twenty-six (6.2%) lay public participants added further sources: personal experience and beliefs, *n* = 10; therapy, *n* = 6 (one participant named EMDR); conversations with friends, *n* = 2; Radio 4 and media *n* = 2, caring for a parent with dementia, *n* = 1. Mental health professional participants also entered further information. Responses were received from 21.7% (*n* = 20) of participants in the non-trauma-focsused group, 24.2% (*n* = 15) of participants in the trauma-focussed EMDR group and 41.7% (*n* = 10) in the trauma-focussed (Other) group. Clinical practice and personal/life experience were most frequently identified as extra sources of knowledge by all three mental health professional participant groups. For sample responses, see the Supplemental materials, p. 16.

### Additional participant comments about memory beliefs

Finally, to better understand the nuances of individual beliefs, and detect common themes between groups all participants were asked: “*If the above questions did not allow you to specify your beliefs exactly, please feel free to comment belo*w”. Over one quarter (26.4%, *n* = 47) of mental health professional study participants wrote comments. The percentage of written responses by the group was: trauma-focussed EMDR, 22.5% (*n* = 14); the trauma-focussed (Other), 50.0% (12) and non-trauma-focussed, 22.8% (*n* = 21). Some broad themes emerged that memories may be inaccurately recorded and recalled; that pre-verbal memories and/or body sensations can be accurately retrieved and verbalised; and that the client’s truth was more important than factual accuracy. For example, one EMDR practitioner operating solely in the private sector – also a hypnotherapist and psychologist – and a member of the British Psychological Society and EMDRUK, chose “Strongly agree” for both repression and dissociative amnesia and wrote:

I believe all memories are stored, but not always accurately. In a sense this doesn’t matter when we work with memories as it is the belief about the memory that impacts the client’s belief about their selves and the world which is important. So we can work with a memory that isn’t fully accurate. Regarding false memories - I think these are very unlikely.

Another EMDR practitioner, clinical psychologist, and member of the British Psychological Society and EMDRUK (working in the NHS and private sector) chose “Disagree” for repression and “Agree” for dissociative amnesia and wrote:

Memory is much more complicated than accurate exact recall of what happened in conscious awareness. With memories of events in the first years of life I think it’s unlikely most people will have conscious memories but likely they do have implicit/body-based memories.

A study participant from the non-trauma-focussed group – a human givens and psychotherapist practitioner – chose “Agree” for repression and “Strongly agree” for dissociative amnesia wrote:

Traumatic memories can be repressed and can be restored. Any recall experience is the most recent version of the event. Our memories are affected in some way by our experiences and other external input. Early pre-verbal experiences can be retrieved and verbalised later especially if triggered by a sensory experience.

For all professional participants’ comments, see Supplemental Materials Table S11, p. 22; for lay public participants, see Radcliffe and Patihis (2024), Supplemental Materials.

### Survey section 3 for all mental health professional participants

Section 3 replicated a survey of American Board-Certified psychiatrists (*N* = 301), see Pope et al. (1999). The first question was: “*If the DSM-5 were to be revised today, how should it treat the diagnosis of dissociative amnesia?*” Thirty-three percent (*n* = 7) of participants who responded in the trauma-focussed (Other) group ticked the box for “No opinion”. Whilst in the non-trauma-focussed group, of the 77 participants who responded, 41.6% (*n* = 32) ticked “No opinion” as did 42.1% (*n* = 24) of the trauma-focussed EMDR group. However, trauma-focussed EMDR practitioners were the most accepting of this diagnosis, with 31.6% (*n* = 18) ticking the box, “Include without reservations” compared with 18.2% (*n* = 14) of non-trauma-focussed participants and 9.5% (*n* = 2) of trauma-focussed (Other) participants. For all group responses to this item, see Supplemental Materials Table S9.

The second question was, “*If the DSM-5 were to be revised today, how should it treat the diagnosis of dissociative identity disorder?*” Again, the trauma focussed EMDR practitioners showed the highest percentage of unreserved acceptance for this disorder; 43.9% (*n* = 25) selected “Include without reservations”. See Table 7 for all study group responses, and Pope et al’s. (1999) survey responses for comparison.

The third question was “*In your opinion, what is the status of scientific evidence regarding the validity of dissociative amnesia*?” Nearly a quarter 24.6% (*n* = 14) of EMDR practitioners who responded to the survey question chose, “Strong evidence of validity” – the highest endorsement by far of all three groups – also exceeding Pope’s American psychiatrists (1999). However, over 42% of participants (who responded) in all three groups selected “No opinion”. See Supplemental Materials Table S10 for all study group responses to this question.

The fourth of Pope et al’s (1999) survey questions asked, “*In your opinion, what is the status of scientific evidence regarding the validity of dissociative identity disorder*?” Here again, the trauma-focussed EMDR practitioners showed the highest endorsement for scientific validity with 36.8% (*n* = 21) selecting “Strong evidence of validity” – exceeded Pope et al’s (1999) American psychiatrists (21%, *n* = 62). However, 36.8% (*n* = 21) of EMDR practitioners also selected “No opinion”. No EMDR practitioner selected “Little or no evidence of validity”. See Table 8 for responses from all study groups.

**Table 8.** Comparing group beliefs for the scientific status of dissociative identity disorder.

Participant Group	Item question: In your opinion, what is the status of scientific evidence regarding the validity of dissociative identity disorder?				
	*n*	Little or no evidence of validity % (*n*)	Partial evidence of validity % (*n*)	Strong evidence of validity % (*n)*	No opinion % (*n*)
Non-trauma-focused	78^1^	7.7 (6)	34.6 (27)	10.3 (8)	47.4 (37)
Trauma-focused EMDR	57	0.0 (0)	26.3 (15)	36.8 (21)	36.8 (21)
Trauma-focused (Other)	21	19.0 (4)	28.6 (6)	19.0 (4)	33.3 (7)
American Psychiatrists (1999)	301	20.0 (59)	51.0 (153)	21.0 (62)	9.0 (27)

Note. This table is adapted from, “Attitudes Toward DSM-IV Dissociative Disorders Diagnoses Among Board-Certified American Psychiatrists” Pope et al., 1999, *American Journal of Psychiatry*; the data from this study population (Board-certified psychiatrists) was included for comparison purposes. ^1^ This group had the highest number of missing cases; 15.2% (14). Participant groups are described in Table 2.

#### Participants’ clinical experience of dissociative identity disorder

Mental health professionals were also asked: “*How many cases have you dealt with that met the criteria for a diagnosis of dissociative identity disorder (DID) since 2010?*” In the non-trauma-focused group 79.3% (*n* = 73) of participants responded to this question. Of those who responded, 53.4% (*n* = 39) had not “dealt with” (seen) any such case and 46.6% (*n* = 34) had seen one or more case: M = 3.55; SD = 12.7; range = 96 [0–96]. Forty-one percent (*n* = 30) had seen between 1 and 5 cases and 5.6% (4) between 25 and 96 cases: In the trauma-focussed EMDR group, 85.5% (*n* = 53) participants responded. Of these, 34.0% (*n* = 18) had seen no such case and 66.1% (*n* = 35) had seen 1–30 cases: M = 3.17; SD = 6.01; range = 30 [0–30]. Twenty-two percent (*n* = 12) had seen 1 case; 13.2% (*n* = 7) 2 cases; 7.5% (*n* = 4) 3 cases;1.9% (*n* = 1) 4 cases; 5.5% (*n* = 3) 6, 7, and 8 cases respectively and 5.7% (*n* = 3) had seen 5 cases and 5.7% (*n* = 3) had seen 10 cases. Two outliers (3.8%) had seen 30 cases: In the trauma-focused (Other) group, 87.5% (*n* = 21) of participants responded. Of these, 38.1% (*n* = 8) had not seen any such case and 61.9% (*n* = 13) had seen one or more cases: M = 2.81; SD = 3.37; range = 12, [0–12]. Fifty-two percent (*n* = 11) participants had seen between 1 and 5 such cases and 9.6% (*n* = 2) had seen 10 and 12 cases each.

Outlier participants in the non-trauma-focused and trauma-focused EMDR groups who had “dealt with” 25 or more cases (since 2010) that met the criteria for DID were further explored. In the non-trauma-focussed group, four participants had seen 96, 40, 30 and 25 DID cases, respectively, and were >57 years of age. Three participants identified as hypnotherapists and one as a human givens practitioner (but was also a member of the National Hypnotherapy Society.)

Within the trauma-focused EMDR practitioner group, two outlier participants claimed 30 DID cases each. One participant aged 61, selected eight professional titles, including hypnotherapist, trauma-informed therapist and neurolinguistic programmer. The another participant, also a female practitioner, was aged 47 years and identified as a clinical psychologist, internal family systems and trauma-informed therapist. Neither of these two EMDR participants had handled a case of satanic ritual abuse. See Supplemental materials, pp.19–20 for further information on participants reporting high numbers of DID cases.

#### Participants’ clinical experience with cases featuring satanic ritual abuse

Mental health participants were also asked, “*How many cases of satanic ritualistic abuse have you dealt with since 2010*”. In the non*-*trauma-focussed group, 81.5% (*n* = 75) of participants responded. Of these, 85.3% (*n* = 64) had not seen such a case; 8.0% (*n* = 6) had seen one case and 6.5% of participants (*n* = 5) had seen 2, 3, 5, 9 and 10 cases, respectively. In the trauma-focussed (other) group, 87.5% (*n* = 21) of participants responded. Of these, 71.4% (*n* = 15) had not seen any such case. Nineteen percent (*n* = 4) had seen one case, and 9.5% ( =  2) had seen 2 such cases. In the trauma-focussed EMDR group, 85.5% (*n* = 53) of participants responded. Of these, 71.7% (*n* = 38) had not seen any such case; 20.8% (*n* = 11) had seen one case, and 7.6% (*n* = 4) had seen between 2 and 6 cases.

#### Beliefs about blocked trauma memories, medically unexplained symptoms/PNES and child sexual abuse

Mental health professional participants were asked two further questions copied from Kemp et al. (2013). Note that the diagnostic descriptors, medically unexplained symptoms and PNES are interchangeable. Participants who agreed to some extent, are shown in Table 9. Trauma-focussed EMDR practitioners showed the highest percentage of agreement for both statements. Fifty-nine percent of EMDR practitioners (*n* = 34) unequivocally “Agreed” that “blocked out” memories can result in physical symptoms such as non-epileptic seizures (PNES), compared with 45% (*n* = 10) of trauma-focussed (Other) practitioners and 34.6% (*n* = 27) of non-trauma-focussed practitioners. In contrast, 31.7% of trainee clinical psychologists (*N* = 375) surveyed in 2012, unequivocally agreed with this statement (Kemp et al., 2013).

**Table 9.** Group beliefs about blocked memories, childhood sexual abuse and medically unexplained symptoms.

	Non-trauma- focused *n* = 78/79^1^	Trauma – focused (Other) *n* = 22	Trauma-focused EMDR *n* = 57	Clinical psych. (2013) *n* = 375
Item 1	Memories of past trauma that are inaccessible to conscious memory or “blocked out” can cause an alteration in a person’s consciousness/dissociation and result in physical symptoms such as altered motor function or non-epileptic seizures			
Percentage of participants who “somewhat agreed” or “agreed”	78.2 (61)	81.9 (18)	91.2 (52)	74.1 (277)
Item 2	In your opinion, what degree of risk does childhood sexual abuse present for developing medically unexplained symptoms			
Percentage of participants who agreed there was a “medium” or “high” risk	88.6 (70)	90.9 (20)	94.8 (54)	65.7 (246)

Note. These item statements originate from Kemp et al. (2013). The study responses from trainee clinical psychologists in Kemp’s survey are shown in the far right-hand column. The total number of participant responses for items 1 & 2 above in each study group varied due to missing cases. ^1^For item statement 1, *n* = 78 and for item 2, *n* = 79.

Concerning the degree of risk posed by child sexual abuse for developing medically unexplained symptoms (PNES), 77.3% (*n* = 17) in the trauma-focussed (Other), 73.7% (*n* = 42) of trauma-focussed EMDR and 63.3% (*n* = 50) of non-trauma-focussed practitioners believed this posed a high risk. In contrast, 30.3% of trainee clinical psychologists surveyed in 2012 believed this posed a high risk (Kemp et al., 2013).

## Discussion

We surveyed 178 UK mental health professionals about their beliefs regarding autobiographical memory function for ordinary and traumatic experiences. We captured new data on mental health practitioners’ beliefs for: unconscious repression, dissociative amnesia, dissociative identity disorder (DID) and causes of psychogenic non-epileptic seizures (PNES), a prevalent, functional, neurological disorder.

Our main research questions were: What proportion of UK mental health professionals believe in repression and dissociative amnesia and do the beliefs of non-trauma-focussed practitioners, trauma focussed EMDR practitioners and trauma-focussed (Other) practitioner participants vary? We found all three study groups showed high agreement for unconscious repression, item 5, (>78%) and increased agreement for dissociative amnesia, item 9 (>84%). The trauma-focussed (Other) and trauma-focussed EMDR practitioner participant groups showed the highest agreement for these two notions: for unconscious repression (91%, 82%) and dissociative amnesia (96%, 90%), respectively, agreed to some degree. Moreover, the trauma-focussed (Other) participant group showed the highest degree of agreement for the accurate therapeutic retrieval of repressed memories, item 6 (75%) and hypnotic retrieval of hidden memories, item 7 (55%). Trauma-focussed (Other) study participants also showed the highest degree of scepticism for false memory development, item 8; 26% disagreed or somewhat disagreed with this phenomenon, compared with 16% of trauma-focussed EMDR participants and 12% of participants in the non-trauma-focussed study group.

Our other survey aim was to better understand mental health professionals’ beliefs about DID because dissociative amnesia for childhood trauma underpins this diagnosis (see Brand et al., 2014; DSM-5-TR, pp. 331–332). In our study, a novel finding was that participants in the trauma-focussed EMDR group showed markedly higher beliefs for the diagnostic validity of this disorder; 37% of trauma-focussed EMDR participants believed it had strong scientific validity compared with 19% of participants in the trauma-focussed (Other) group and 10% in the non-trauma-focussed group. Relatedly, trauma-focussed EMDR participants were nearly twice as likely as other mental health professional participants, to unreservedly endorse the diagnostic inclusion of DID.

Finally, other novel findings were, a high degree of agreement amongst all three professional groups that childhood sexual abuse poses a medium or high risk for developing medically unexplained symptoms (>88%) and that “blocked out” trauma memories are causally related to dissociation and psychogenic non-epileptic seizures (PNES; > 78% somewhat agreed or agreed). Again, participants in the trauma-focussed EMDR group showed the strongest degree of belief in these items. We now review the implications of survey findings by study group.

### Trauma-focussed EMDR practitioners

A large majority of EMDR participants showed scientific misconceptions about memory function for traumatic experiences that some scholars consider potentially harmful to public health (Lilienfeld, 2007; McHugh, 2008; Otgaar et al., 2021b; Paris, 2023; Yapko, 1994b). Most EMDR participants endorsed repression, a concept that is scientifically questionable (McNally, 2005; Otgaar et al., 2019; Shobe & Kihlstrom, 1997) and nearly all, (90%) endorsed dissociative amnesia – arguably, an equally controversial concept (Lynn et al., 2014; Mangiulli et al., 2022; Otgaar et al., 2025b; Patihis, 2023) and analogous with repression (see Otgaar et al., 2019). Despite the relatively small sample size, our study findings of high belief in repression and dissociative amnesia align with recent international (Houben et al., 2021a, 2021b; Mangiulli et al., 2021; Otgaar et al., 2025a; Zappalà et al., 2024; Wessel, 2018). Concerningly, 34% of EMDR practitioners chose “don’t know” or disagreed to some degree that false memories were possible (survey item 8) compared with 40% of the trauma-focussed group and 29% of the non-trauma-focussed group.

A non-trivial minority of EMDR practitioners also showed a flawed scientific understanding for non-traumatic, everyday memory function. For example, for the incorrect notion that the mind accurately records events as they happen, 17% of EMDR participants slightly or strongly agreed. Moreover, 39% agreed that memories are accurately stored and retrievable from the first year of life – an appreciably higher percentage than lay public participants (28%) – echoing psychotherapists’ beliefs (40%) surveyed by Yapko (1994a) at the zenith of the memory wars (Crews, 1995).

Our study findings show that many EMDR participants held beliefs that were in keeping with mainstream scientific understanding, but some did not, and sometimes these beliefs conflicted with one another. For example, 96% of EMDR practitioners correctly agreed to some degree in the reconstructive, malleable nature of memory, yet 50% also inaccurately agreed in the permanence of memory. Moreover, over half (56%) believed in the accurate therapeutic retrieval of repressed memories and 38% in accurate hypnotic retrieval. Houben et al. (2021a, 2021b) also found conflicting accurate and inaccurate scientific beliefs amongst their EMDR study group. For instance, whilst 97% of their EMDR practitioner participants correctly rejected the metaphor comparing memory with a video recording, 63% also paradoxically believed, “the body may remember trauma outside of the mind’s awareness”.

We also compared our trauma-focussed EMDR study group’s scores with US clinical psychologists’ beliefs surveyed by Patihis et al. (2014). Our participants’ scores were higher (i.e., less sceptical) than U.S. clinical psychologists for survey items relating to memory permanence, repression, accurate therapeutic retrieval, and accurate hypnotic retrieval, see Table 3 above. Concerningly, in a later study (Patihis & Pendergrast, 2018), ten participants named EMDR as a therapy that “they knew involve[d] repressed memories” p. 12.

#### Beliefs associated with the scientific validity of DID and dissociative amnesia

We examined beliefs for DID and dissociative amnesia, replicating survey items from Pope et al. (1999) who surveyed American psychiatrists’ beliefs. Our trauma-focussed EMDR study group gave the strongest endorsement (of all three study groups) for the unreserved diagnostic inclusion of DID (44%). In contrast, only 35% of American psychiatrists endorsed this (Pope et al., 1999). However, for this same item, 28% of EMDR participants selected, “No opinion” compared with 9% of American psychiatrists (Pope et al., 1999).

Interestingly, endorsement for the unreserved diagnostic inclusion of dissociative amnesia was lower; only 32% of EMDR agreed with this – comparable with American psychiatrists in 1999 (35%) (Pope et al., 1999). Interestingly, 42% of EMDR participants chose, “No opinion”, compared with 9% of American psychiatrists (Pope et al., 1999). Interpreting EMDR participants’ “No opinion” responses is difficult, especially given the very high degree of agreement to dissociative amnesia (survey item 9). This may indicate that EMDR practitioners have merely learnt that dissociative amnesia exists but have limited scientific understanding. Still, a non-trivial 24% of participants in the EMDR study group believed that there was strong scientific evidence for dissociative amnesia, again, the highest endorsement of all three groups.

**Clinician experience of DID.** EMDR practitioners’ clinical experience of DID was also the highest of all three participant groups. Two-thirds of responding participants (66%; 35) had “dealt with” at least one case of DID since 2010, and of these, 9% had seen 10–30 cases, M = 3.17. In our study, the prevalence of DID cases seen by EMDR participants was markedly higher than the 40% of study participants in Ost et al. (2013) – which they considered “surprising” (p. 13). The increased prevalence rate in our study may indicate improved diagnostic detection of this complex and controversial disorder and/or that the clinical incidence of DID is increasing. Alternatively, it may indicate that some mental health practitioners are misdiagnosing or overinterpreting dissociative symptoms. Overall, we consider that our EMDR study findings are concerning.

#### Beliefs associated with PNES (medically unexplained symptoms)

Participants were firstly asked about their agreement for the existence of a *causal* relationship between unconsciously “blocked” trauma memories and physical symptoms such as non-epileptic seizures (PNES). This idea is linked with the controversial theory of body memories (Van der Kolk, 1994, 2015) and also aligns with the EMDR AIP model (Shapiro, 2010, 2014, 2018). Perhaps unsurprisingly, the trauma-focussed EMDR group showed the highest agreement; (91%) somewhat agreed or agreed. All groups exceeded clinical psychologists’ surveyed by Kemp et al. (2013). Secondly, participants were asked directly about the degree of risk that childhood sexual abuse posed for the development of unexplained medical symptoms (PNES). Again, EMDR practitioners believed that childhood sexual abuse posed a greater degree of risk (95%). Such beliefs, when considered alongside the EMDR AIP theory, memory recovery techniques (e.g., the floatback), lay patient belief in traumatic repression/dissociation of memories and high motivation by the therapist and patient to find an explanation for current problems, may elevate the risk of false memory development. Importantly, Shapiro also advises EMDR practitioners that “Chronic abuse is a leading cause of dissociative disorder … ” (Shapiro, 2018, p. 303).

It is unarguable that childhood trauma of any kind can lead to psychological disturbances and psychiatric disorders, (Hailes et al., 2019). However, we argue that the severity, uncertain etiology and high risk of self-harm associated with patients suffering from these dissociative disorders warrants a careful, empirically supported, treatment response. In the UK, mental health professionals in the private sector comprise a diverse array of clinical and non-clinically oriented professionals. They are permitted to treat many complex psychological conditions, including DID and PNES. However, there is no current UK data (to our knowledge) on the prevalence of private treatment (compared with public treatment) for these disorders, the professional titles and clinical expertise of treaters, or adverse treatment outcomes (but see Lewis et al., 2020, dropout measures for PTSD).

#### EMDR as a treatment for PNES and DID

Some scholars have advocated EMDR therapy for PNES (Cope et al., 2018; Van Rood & De Roos, 2009). However, whether UK EMDR practitioners are treating PNES patients is unknown. Arguably, EMDR practitioners holding contentious memory beliefs may pose an elevated risk of harm to PNES (and DID) patients. They may misinterpret or overinterpret patients’ dissociative symptoms, leading to flawed treatment, flawed diagnoses and memory recovery for non-experienced events. For example, pelvic thrust movements – often exhibited during seizures (Vijay & Reuber, 2024) – may be misinterpreted as somatic symptoms of unprocessed trauma memories. Moreover, controversial public information about PNES may reinforce therapists’ (and patient) prior belief in repression e.g., that seizures represent trauma stored in the body, “expressing what the mind and mouth cannot” (Epilepsy Foundation website, October 13, 2019). This site also recommends EMDR as the most effective treatment. Similarly, a UK website suggests that seizures may be the body’s way of “drowning out” or “unconsciously repress[ing]” painful memories (Epilepsy Society, 2023). EMDR treatment of DID patients raises similar concerns.

For the treatment of DID, our data show many EMDR study participants handled patients with DID. However, scant research exists on EMDR treatment prevalence or outcomes for patients with DID. We posit that patients diagnosed with DID or PNES comprise an especially vulnerable patient cohort. Such patients who seek EMDR treatment with no prior history of trauma or maybe suspect they may have either condition may be more vulnerable to adverse health outcomes, e.g., false memories and increased psychological distress.

Increased mental distress is a known side-effect for all patients receiving EMDR memory treatment (EMDR UK; NHS, 2023). Practitioners are trained to handle this (Roth et al., 2021). Whether increased distress is more associated with newly recovered memories (that may not reflect experienced events) is unknown. Recent research shows that EMDR has a non-trivial mean patient dropout rate; 18% for EMDR, compared with 9% for behavioural therapy for treating PTSD (Lewis et al., 2020; but see Wright et al., 2024); reasons for EMDR dropout are unclear. Another research concern is the combined (potentially adverse) effect of the Adaptive Information Processing (AIP) model with practitioners’ prior beliefs for repression or dissociative amnesia.

New memory recovery during EMDR treatment is also an expected therapeutic outcome. Shapiro (EMDR’s founder) highlights the likelihood of new *and accurate* memory recovery (Shapiro, 1989a, 1989b, 2001, 2018). She relates, “Many true memories will surface for the first time … ” (Shapiro, 2018, p. 302). Yet elsewhere she cautions, “there is no way of knowing whether a memory that emerges is true … (p. 299).” She also exhorts clinicians to be aware of the potential for memory distortion and to minimise this risk by not discussing details or interpreting memories that surface (p. 302). In another section, “Appropriate goals” (p. 293), she warns clinicians that the accurate retrieval of memories of abuse or forgotten childhood events, is “questionable”. Whether Shapiro’s cautionary words above are included in current training and how they translate in practice is unknown. EMDR protocols and training content vary.

Recently, attention has been drawn to the “proliferation” of international protocols and varying techniques (Laliotis et al., 2021, p. 189). Differences in training content and delivery methods may affect treatment outcomes, e.g., increase the potential for inadvertent false or distorted memory recovery via dissociative “flashbacks” or patient dropout.

In the UK, the National Institute for Health and Care Excellence (NICE, 2018) recently raised (unspecified) concerns about inconsistency in EMDR delivery (see recommendations 1.6.18–20). A UK EMDR competency framework has now been published (see Roth et al., 2021). This framework endorses the AIP model and underlines the strong link between dissociation and prolonged chronic trauma. It explains that fragmentation of personality (i.e., DID) can occur in extreme cases (Royal College of Psychiatrists, 2021a). The framework also approves the floatback technique (Browning, 1999) for patients diagnosed with Complex PTSD (C-PTSD; ICD-11, 2018; Royal College of Psychiatrists (2021c)). Yet, such patients often have complex psychopathology (Paris, 2023) including depression and high levels of self-harm (DSM-5-TR). Moreover, all EMDR patients, prior to desensitisation, receive psychoeducation based on the AIP model of memory for trauma.

Lastly, therapists and/or vulnerable patients may start therapy, already presuming hidden memories of trauma exist, that await therapeutic discovery to bring psychological relief (Rubin & Boals, 2010). Multiple converging factors, including contentious memory beliefs (held by the therapist and lay client), postgraduate clinical or therapeutic education and EMDR theory may potentiate the risk of adverse outcomes e.g., memory distortion for non-experienced events.

### Trauma-focussed (Other) group

Study findings revealed that these participants held the most accurate scientific beliefs for item 1, the mind is like a computer, 92% disagreed to some degree, and for item 3, the reconstructive nature of memory, 96% agreed to some degree. Yet simultaneously, this group also held the most *inaccurate* beliefs of all three groups for: repression, (92% agreement), accurate therapeutic retrieval (75% agreement), hypnotic retrieval (55% agreement) and dissociative amnesia (96% agreement). Interestingly, this group showed stronger belief for repression than dissociation (37% and 21% respectively chose, “strongly agree”). These inconsistent beliefs highlight a trend seen in other studies (e.g., Houben et al., 2021a; Patihis et al., 2014). They signal a dual belief system whereby some beliefs align with mainstream scientific thinking and some do not.

Notably, three-quarters of this study group also believed that repressed memories could be accurately therapeutically retrieved – a markedly higher score than the other two study groups. This begs the question, “How can health professionals believe in repression or dissociation and accurate therapeutic retrieval and simultaneously believe that memory is impermanent, reconstructive and malleable?”

Arguably, the most likely explanation for these dissonant beliefs is professional (mis)education about traumatic memory function. Many varying psychoanalytical theories and practices coexist in the UK and elsewhere (Paris, 2023). Some, psychotherapies, e.g., internal family systems (IFS) and neurolinguistic programmers termed, “alternative therapists” (Patihis et al., 2014) teach non-mainstream theories about memory and cognition. Past and current research indicates such therapists may be less scientifically informed (Murphy et al., 2025; Paris, 2023; Patihis et al., 2014; Pignotti & Thyer, 2015).

In this study group, IFS therapists were the most represented professional title, accounting for 62% (15) of the entire group, followed by trauma-informed therapists accounting for 54% with some overlap between these titles. IFS theory of personality and memory is scientifically contentious. It claims there is a “core positive Self” (See Pignotti & Thyer, 2015, p. 197) with subpersonalities protecting this core self from past negative experiences. Whether IFS therapists’ beliefs pose an elevated risk of adverse health outcomes (e.g., eliciting recovered memories for non-experienced events) is unknown. Moreover, a potential study limitation is that the beliefs held by our small sample of IFS therapists (*n* = 15) may not be representative of all UK IFS therapists. It is noteworthy that in our study, IFS therapists comprised 18% (11) of EMDR practitioners (see Supplemental Materials, Table S4). We also acknowledge that this study group does not represent the diversity of trauma-focussed therapists (see Schnurr, 2017).

Concerningly, recent research examining adverse therapeutic outcomes for PTSD, i.e., dropout rates, showed a significant association between trauma-focussed therapies and increased dropout rate (Lewis et al., 2020). Our study findings may be a tentative indication that some trauma-focussed psychotherapists’ beliefs pose an increased risk to vulnerable patients. We conclude further research attention on trauma-focussed therapists’ beliefs and memory recovery practices is justified.

### Non-trauma-focussed group

This study group had the largest number of participants (*n* = 92). The most frequently selected professional titles were hypnotherapist, 27% (25), clinical psychologist, 26% (24) and then psychotherapist, 24% (22). Overall, non-trauma-focussed participants were more sceptical towards the notions of repression and dissociative amnesia compared with the other two study groups. The majority in this participant group agreed or strongly agreed in repression and dissociation – 54% & 52%, respectively. However, overall, clinical psychologists were more sceptical than hypnotherapists. No clinical psychologist participant strongly agreed with either notion. Increased scepticism by these clinical psychologist participants aligns with past research (Ost et al., 2013; Patihis et al., 2014). Clinical psychologists’ degree of belief in repression echoed past findings. In Patihis et al. (2014), 60% of US clinical psychologists agreed to some degree compared with 58% of our UK participants. We suggest this is still a high percentage. Clinical psychologists’ belief in dissociative amnesia was measured for the first time in this study. This subgroup showed increased belief in dissociative amnesia compared with repression; 50% and 29% respectively agreed or slightly agreed that dissociative amnesia prevents a person from recalling traumatic experiences (Table 6).

Hypnotherapists’ beliefs were especially questionable in the context of false memory recovery and DID cases. We posit a non-trivial minority may pose an increased risk towards vulnerable patients. Of concern were four participants – three hypnotherapists and one Human Givens – handling 96, 40, 25 and 30 cases of DID respectively; interpreting these figures in isolation is problematic. Further research on hypnotherapist training and practice is warranted. In sum, our data aligns with past study findings that non-clinical (arguably, non-mainstream) therapists may pose an elevated risk of adverse outcomes in therapy (Ost et al., 2013, 2017; Patihis et al., 2014; Patihis & Pendergrast, 2018).

### Study implications

Adverse treatment outcomes are a known risk factor of psychotherapy (see Barlow, 2010 for a historical overview). Understanding factors associated with adverse therapeutic outcomes is a growing research concern (e.g., Crawford et al., 2016; Klatte et al., 2018; Scott & Young, 2016). Iatrogenic false memory induction for non-experienced events (e.g., childhood abuse) is a recognised, adverse outcome (Lilienfeld, 2007) with real-life consequences for patients, their families and third parties (Lambert & Lilienfeld, 2007; Li et al., 2023; Otgaar et al., 2022; Pendergrast, 2021). Contentious memory beliefs play a key role in the development of false memories (Lynn et al., 2015, 2023; McNally, 2003; Yapko, 1994b).

Understanding the sources of such beliefs is essential knowledge for reframing beliefs, reducing adverse outcomes and safeguarding public health. Significantly, most of our study participants (>90%) chose “professional education” as their knowledge source (for memory beliefs). Moreover, “innate beliefs” were chosen by 33% of study participants in the trauma-focussed group and 63% of the lay public (the highest score). Future research might investigate precisely what various mental health professionals are being taught about memory and cognition.

Our data show that UK mental health professionals are a heterogeneous group, from diverse clinical and non-clinical backgrounds. Educational content will vary between professional groups. Notably, our trauma-focussed study participants were mainly private sector therapists where alternative theoretical ideas and problematic memory beliefs may be more plentiful. The ascendance of the posttraumatic model (Otgaar et al., 2019) may partly explain the increased belief in dissociative amnesia in our study sample. We posit this model may underpin current UK mental health and trauma education (e.g., Mind, 2023, UK public guidance on dissociative disorders) and trauma-informed guidance. Consequently, mental health professionals may be unaware that the notion of dissociative amnesia and implicit memory storage is scientifically contentious (see McNally, 2003, pp. 178–189 for a rebuttal of implicit body memory theory).

Therapists’ understanding of false memory research may also be limited (Murphy et al., 2025). Our data indicates that some mental health professionals are aware of false memories and some are uncertain. We question whether modern-day clinicians might wrongly perceive the lack of evidence of proven false memories, as evidence of their absence. Further, they may wrongly believe false memories can only be induced by direct suggestion (see Patihis & Herrera, 2024, memory modification of emotion without suggestion; see Otgaar et al., 2017 sponataneous false memories and associative-activation theory).

### Future research

We confine our recommendations to EMDR theory and practice. Understanding EMDR practitioners’ therapeutic orientation and clinical education is key to understanding their beliefs and practices. Further research might try to capture a more nuanced understanding of practitioners’ memory beliefs and practices and collect clinical data, e.g., patients’ presenting diagnoses, prior symptoms, treatment length and recorded adverse outcomes. Finally, we highlight that future research might examine the scientific validity of the AIP model and the scientific accuracy of psychoeducation given to patients and examine the prevalence and methodology for eliciting new (negative) memory recovery prior to bilateral stimulation.

In the past, the second author and other scholars have discussed the benefits of informed consent to mitigate against the risk of false memory recovery. We now consider this step might be insufficient, as memory misunderstanding may be too deeply embedded. We reflect that an educational outcome of the memory wars was the issuing of scientific guidance for psychologists and psychiatrists (See Supplemental materials, p. 30). This guidance explained the adverse therapeutic consequences of belief in repression and associated memory recovery practices; it has since lapsed or been withdrawn. We suggest new international guidance for mental health professionals is required. This could outline current scientific issues on memory for trauma (disputed and agreed) and highlight questionable therapeutic beliefs and practices that might elicit false, harmful memories.

### Study limitations

The current study has several limitations. Firstly, our study sample is unrepresentative of UK mental health professional titles, e.g., only six psychiatrists participated. Understanding psychiatrists’ memory beliefs is important given their enhanced clinical status and expert role in justice settings; in our study, four psychiatrist participants were identified as experts (See Supplemental Materials Table S8 for experts’ survey responses). Moreover, our study sample is unrepresentative of the heterogeneity of UK mental health professionals and the prevalence of specific therapy types, e.g., only 14% (25) of study participants identified as cognitive behavioural therapists. Secondly, study participants may have, inadvertently, been allocated to the wrong group, e.g., to the non-trauma-focussed study group instead of the EMDR trauma-focused group. Group allocation depended on participants’ accurate choice of professional titles. Some participants may have overlooked ticking a title, or preferred using one descriptor over another, e.g., some clinical psychologist participants did not select “EMDR practitioner”. However, a subsequent inspection of optional comments and professional membership suggested they might be EMDR practitioners.

Other considerations concern potentially ambiguous survey wording and Likert responses that may misrepresent the nuances of participants’ beliefs (see Brewin, 2021; Brewin et al., 2019; but see Otgaar et al., 2020). However, our participants’ responses for key survey items align with previous and current research. We therefore conclude that study integrity is not undermined. Overall, despite some limitations, our study sheds new light on UK mental health professionals’ beliefs, especially EMDR practitioners. We hope it will provide a stepping-stone for further research on mental health professionals’ education, memory beliefs, practices and adverse outcomes in psychotherapy.

## Conclusion

Our overarching study aim was to measure UK mental health professionals’ beliefs for repression and dissociative amnesia and consider their potential impact on adverse health outcomes especially, false memory induction. We captured new data for EMDR practitioners’ memory beliefs and practices. Overall, our findings indicate trauma-focussed mental health professionals have elevated beliefs for controversial memory notions when compared with non-trauma-focussed professionals. Our data also indicates that overall belief in dissociative amnesia is more prevalent than belief in repression. We spotlighted aspects of EMDR theory, training and treatment that may pose an increased risk for new memory recovery for non-experienced events.

Lessons from the past may need to be relearnt and new guidance created for younger professionals unfamiliar with the suffering caused by the Memory Wars. Memory scholars must continue to progress evidence-based scientific knowledge that may not be welcomed by some but is essential for the public good.

## Supplementary Material

Supplemental Material

## Data Availability

The data that support the findings of this study are openly available in redacted form in the Zenodo repository at **10.5281/zenodo.14833229.** Data that may potentially identify participants have been removed.
