# Supplementary material for: In a UK sample, EMDR and other trauma therapists indicate beliefs in unconscious repression and dissociative amnesia
Source: Memory. 2025 May 11;33(5):542–65. doi: 10.1080/09658211.2025.2498929 (PMC12309458; doi:10.1080/09658211.2025.2498929)
Supplement: Supplemental Material [file PMEM_A_2498929_SM5801.docx]

**In a UK Sample, EMDR and Other Trauma Therapists Indicate Beliefs in Unconscious Repression and Dissociative Amnesia**

**Supplemental Online Material**

Pamela J. Radcliffe and Lawrence Patihis

Department of Psychology, University of Portsmouth, United Kingdom

**Supplemental Introduction**

**Evidence of increased academic and clinical interest in dissociation**

Between 2010–2019, scholars located 71 articles concerning dissociative amnesia in a dedicated trauma journal, compared with no such publications between 1990–1999 (Otgaar et al., 2019; and see Dodier, 2019). Professional organisations e.g., the European Society for Trauma and Dissociation (ESTD; <https://estd.org/>) educate mental health professionals about trauma related dissociative disorders according to the posttraumatic memory model. Within the UK, specialist clinics for dissociative disorders now exist e.g., the Clinic for Dissociative Studies (<https://clinicds.org.uk/>) and the Pottergate Centre -The Centre for Trauma and Dissociation (<https://dissociation.co.uk/>. The public website of a leading UK mental health charity, Mind (<https://www.mind.org.uk/>) explains dissociative disorders, including dissociative identity disorder. It describes dissociation as a “natural response to trauma” (Mind, 2023) and describes the potential for dissociation and subsequent amnesia for trauma experienced during childhood. Also see the National Health Service guidance on dissociative disorders, (NHS, 2023). For a historical review of dissociation treatment in the UK from a posttraumatic perspective, see Aquarone & Hughes, (2013); for a skeptical clinical appraisal of UK treatment of dissociative disorders see Mair, (2013). Still, dissociative amnesia remains a scientifically contentious idea, and subject to scholarly debate.

**Further detail on Yapko’s survey results**

In 1992, Yapko (1994) captured novel data from 860 US psychotherapists. He devised two questionnaires to measure psychotherapists’ attitudes towards memory and hypnosis. In the Memory Attitude Questionnaire, for item 2, (measuring belief in repression): “Events that we know occurred but can’t remember are repressed memories – i.e., memories that are psychologically defended against” 21.6% (187) of participants agreed strongly and 37.7% agreed slightly. Nearly one-third of respondents (30.7%) agreed that “When someone has a memory of a trauma while in hypnosis it objectively must have occurred” – a scientifically inaccurate statement. Over a half of respondents (53.8%) believed “Hypnosis can be used to recover accurate memories of past lives” – also, an inaccurate statement. Nevertheless, over three-quarters of respondents (79.2%) also agreed “It is possible to suggest false memories to someone who then incorporates them as true memories.” Yapko considered that the unquestioning acceptance of clients’ new narrative truths was misplaced, despite therapists’ “benevolent intentions” (Yapko, 1994, p. 55)

**Supplemental Method**

**Participants**

Ethnicity for the two target study populations are shown in Table S.1.

**Table S.1** Ethnicity of Lay Public and Mental Health Professional participants

| Ethnic Group | Lay public  *N* = 419 | | Mental health Professionals  *N =*178 | |
| --- | --- | --- | --- | --- |
|  | *n* | % | *n* | % |
| White | 380 | 90.7 | 157 | 88.8 |
| Asian/Asian British | 20 | 4.8 | 6 | 3.4 |
| Mixed/Multiple ethnic groups | 10 | 2.4 | 7 | 3.9 |
| Black/African Caribbean/Black British | 5 | 1.2 | 4 | 2.2 |
| German/Czech Jew | 0 | 0.0 | 1 | 0.6 |
| Arab | 1 | 0.2 | 1 | 0.6 |
| Romany Gypsy | 1 | 0.2 | 0 | 0.0 |
| White European | 1 | 0.2 | 0 | 0.6 |
| Other - unspecified | 0 | 0.0 | 1 | 0.6 |

***Lay public recruitment***

Any British citizen >18 years was eligible to enter the survey. We aimed to capture a minimum of two hundred and fifty participants to provide sufficient study power using G-power. In total, 451 individuals attempted the survey however, 31 cases were removed where survey completion time was < 90 seconds. Lay participants were recruited via the marketing company Prolific <https://www.prolific.co/> and received £1. 64 for taking part. Lay participants by nation: 352 in England, (84.0%); 39 Scotland, (9.3%); 20 Wales, (4.8%); and 8 Northern Ireland, (1.9%). Participants’ socio-economic status was captured by a scale with values between 1-10 depicted by a ladder, where the bottom rung (the lowest status) = 1 and highest rung (highest social status) = 10). Participants’ mostly chose between value 5, 20.8% (*n* = 87) value 6, 23.2% (*n* = 97) and value 7, 18.4% (*n* = 77). Lay participants’ educational attainment ranged from having no academic qualifications 0.5% (*n* = 2) to a doctorate or above, 3.8% (*n* = 16). The most common qualification was a Batchelor’s degree, 37.9% (*n* = 159), followed by ‘A’ Levels or equivalent qualifications, 24.1% (*n* = 101).

***Mental health professional participant recruitment***

Mental health professional participant data was captured in two tranches, firstly between 07/03/2023 – 24/07/2023, then from 29/01/2024 to 25/02/2024. The second tranche comprised solely EMDRUK members (*n* = 30 after filtering out incomplete responses). There were no general medical practitioner participants.

In 2023, 188 mental health professionals started the survey. Participants who completed less than 70% of the survey (*n* = 40) were excluded from study analysis, leaving a sample size of *N* = 148. In February 2024, to improve study integrity for our prime group of interest - EMDR practitioners - we extended data collection. A further advert was placed in the journal of EMDR Association UK – only circulated to members (EMDR practitioners) <https://emdrassociation.org.uk/>. A further 37 study participants agreed to participate and completed the survey. However, seven cases were removed where participants did not identify as EMDR practitioners leaving, *n* = 62 EMDR practitioners/therapists who were included in the study; *N* =178 mental health professional participants overall. Increasing the sub-population of EMDR therapists improved study validity and integrity for separate data analysis of this sample population. EMDR therapists were of special study interest due to their focus on memory processing of traumatic experiences.

Recruitment of Internal Family Systems’ practitioners took place via a generic email containing the study advertisement (and the Qualtrics survey link) that was then emailed to all Internal Family Systems therapists listed in a public directory located on the Internal Family Systems Institute website. The researcher had no direct contact with any mental health professional (prospective participant) about the study or their participation save for one senior psychiatrist who was contacted and asked if would be agreeable to promote the study to his professional group. Details of all professional recruitment are shown in Table S.2.

**Table S.2** Details of Study Mental Health Professional Recruitment

| **Professional body** | **Recruitment details** |
| --- | --- |
| Association of Christians in Counselling and linked Professions | <http://www.acc-uk.org/>  Internal advert, March 2023 |
| Association for Neurolinguistic Programming(NLP) | <http://www.anlp.org> Internal advert, April, 2023 |
| Association for Professional  Hypnosis and Psychotherapy | <https://www.aphp.co.uk/> https://www.[admin@aphp.co.uk](mailto:admin@aphp.co.uk)  Internal advert – emailed to membership & UKcho the UK Confederation of Hypnotherapy Organisations April, 2023 |
| British Psychological Society  (BPS) | <https://www.bps.org.uk/> Internal newsletter emailed survey advert  Division for Neuropsychology – June, 2023; Division for Clinical Psychology – July, 2023 |
| British Association for Behavioural and Cognitive Therapists (BABCP) | <https://www.babcp.com/>  Advert on internal bulletin board research page. 16 03 2023 - 30 05 23 |
| British Association for Counselling and Psychotherapy (BACP) | <https://www.bacp@bacp.co.uk>  Advert on internal research page. Posted 16 03 23 -16 05 23 |
| British Association of Therapeutic Hypnotists | <https://www.bathh.co.uk> Advert placed, April 2023 |
| British Psychoanalytic Council | <https://www.bpc.org.uk> Advert placed, March 2023 |
| Clinic for Dissociative Studies (London) | <https://www.clinicds.org.uk/>Advert circulated internally |
| Counselling and Psychotherapy in Scotland | <https://www.cosca.org.uk/> Advert placed |
| EMDR Association UK | <https://www.emdrassociation.org.uk>  Advert posted in in-house magazine April/May 2023 and reposted in June; Reposted in February, 2024. |
| Human Givens Institute | <https://www.hgi.org.uk> Advert posted on internal social media platform.  Advert posted in members’ Bulletin 24 04 23 for 1 month |
| Internal Family Systems  Institute (IFSI) | <https://www.info@ifstuk>  Advert emailed to membership on Internal Family Systems Directory |
| Portsmouth Hospitals University NHS Trust | Privately circulated survey advert via internal email. |
| Royal College of Psychiatrists | Failed to give final response. But the advert was posted to an internal expert witness group in their newsletter |
| Salisbury Hospital NHS Trust | Advert circulated via internal email to one department |
| UK Council for Pyschotherapy | This is an influential and key professional association for mental health professionals with an 11K membership. It declined to advertise the survey. |

***Further descriptive and professional information***

Mental health participants’ mean years of clinical experience: *M* = 18.64 years; *SD =*11.25 Range, 48 [1 - 49].

Ten study participants (5.6%) identified as expert witnesses. Their professional titles were: Human Givens therapist (*n* = 1), Psychiatrist (*n* = 4), Clinical Psychologist (*n* = 4), Psychotherapist (n = 1). These participants ticked “Yes” to the survey question, “*Do you work as an expert witness*?” Group demographics were as follows; biological sex, females, 50.0% (5), males 50% (5); age, *M*_age_ = 62; *SD* = 8.8; range = 24 [50 - 74], and ethnicity; white, 80.0% (8); Asian/Asian British 10.0% (1); and other ethnic groups, 10.0% (1). Practice location: England, 90.0% (9), Wales, 10.0% (1). Professional titles: Human Givens practitioner (1); psychiatrist, (4); clinical psychologist, (4); psychotherapist, (1). Years of clinical experience, *M* = 34.7; *SE* = 2.7; *SD* = 8.64. Practice domains: NHS, 40% (4); private practice, 90% (9).

In the UK ‘Chartered’ and ‘Clinical Psychologist’ are protected titles however, the title ‘Psychologist’ is not protected, thus any individual may use the title. Moreover, in this study, two individuals chose the title, “Neurologist” however, neither were members of the Institute of Neurologists. Tables S.3 shows the overall frequency of professional titles and Table S.4. compares professional titles within the three study groups. Five mental health professional participants ticked the box that stated they were not required to be registered (i.e. with any professional organisation).

**Table S.3** Percentage Frequency of Professional Titles Amongst All Mental Health Professional Participants

| Mental health professionals’  titles and therapeutic modality | *N*  (178) | % |
| --- | --- | --- |
| Counsellor | 38 | 21.3 |
| Cognitive behavioural therapist | 25 | 14.0 |
| Dissociative disorders specialist | 2 | 1.1 |
| Eye movement desensitization and reprocessing practitioner | 62 | 34.8 |
| Human Givens | 10 | 5.6 |
| Hypnotherapist | 34 | 19.1 |
| Internal family systems | 26 | 14.6 |
| Neurolinguistic programmer | 14 | 7.9 |
| Neurologist | 2 | 1.1 |
| Nurse, mental health | 11 | 6.2 |
| Psychiatrist | 6 | 3.4 |
| Psychologist, clinical | 31 | 17.4 |
| Psychologist, other | 13 | 7.3 |
| Psychotherapist | 54 | 30.3 |
| Trauma informed therapist | 40 | 22.5 |
| Other professional title:  Counselling, EMDR consultant & facilitator, medical device trainer, Psychoanalytic psychotherapist, supervisor, systemic family therapist, systemic psychotherapist, Hypnotherapy trainer. | 9 | 5.1 |
|  | | |

**Table S.4** Participants’ Mental Health Professional Titles by Study Group

|  | Participant groups | | |
| --- | --- | --- | --- |
|  | Non trauma-focused  *n* = 92 | Trauma-focused  (Other)  *n* = 24 | Trauma focused  EMDR  *n* = 62 |
| Professional  therapeutic title(s) | % (*n*) | | |
| Cognitive behavioural therapist | 5.4 (5) | 4.2 (1) | 30.6 (19) |
| Counsellor | 8.7 (8) | 41.7 (10) | 32.3 (20) |
| EMDR therapist | 0.0 (0) | 0.0 (0) | 100.0 (62) |
| Human Givens | 7.6 (7) | 12.5 (3) | 0.0 (0) |
| Hypnotherapist | 27.2 (25) | 12.5 (3) | 9.7 (6) |
| Humanistic therapist | 2.2 (2) | 16.7 (4) | 9.7 (6) |
| Internal Family Systems therapist | 0.0 (0) | 62.5 (15) | 17.7 (11) |
| Neurolinguistic programmer | 8.7 (8) | 12.5 (3) | 4.8 (3) |
| Neurologist | 1.1 (1) | 0.0 (0) | 1.6 (1) |
| Mental health nurse | 0.0 (0) | 4.2 (1) | 16.1 (10) |
| Psychiatrist | 4.3 (4) | 0.0 (0) | 3.2 (2) |
| Psychologist, Clinical | 26.1 (24) | 0.0 (0) | 11.3 (7) |
| Psychologist, Other | 5.4 (5) | 4.2 (1) | 11.3 (7) |
| Psychotherapist | 23.9 (22) | 41.7 (10) | 35.5 (22) |
| Trauma informed therapist | 0.0 (0) | 54.2 (13) | 43.5 (27) |
| Dissociative disorder specialist | 0.0 (0) | 4.2 (1) | 1.6 (1) |
| *Note*. Other professional titles by group : Non-trauma focused (*n* = 3; 3.3%) medical device trainer, supervisor, clinical hypnotherapy trainer; Trauma-focused, (*n* = 1; 4.2%) counselling, psychoanalytic psychotherapist, psychological therapist;  trauma related therapists; EMDR practitioners (*n* = 5; 8.1%) EMDR consultant & facilitator, psychological therapist, systemic family therapist, systemic psychotherapist. | | | |

***Additional descriptive information for groups and subgroups***

***Non-trauma focused mental health professionals***. Group ethnicity overall (*N* = 92) was: White, 87.0% (80); Mixed /Multiple, ethnic groups 6.5% (6); Asian/Asian British, 4.3% (4); Other, one Arab, and one German Czech Jew. Practice location: NI 3.3% (3); Scot, 7.6% (7); Wales, 1.1% (1) ; England 87% (80). Online practice 16.3% (15). The descriptive and professional data of participants who identified as hypnotherapists and clinical psychologists were further explored.

Hypnotherapists (excluding EMDR practitioners) (*n* = 25): *M*_age_ = 54.84; *SD* = 8.9; range = 29 [38-67]; females, 76.0% (19); males 24% (6). Practice type: NHS 12.0% (3); Private sector, 96.0% (24). Other therapeutic titles: Cognitive Behavioural Therapist 12% (3); Counsellor 20% (5); Neurolinguistic programmer 16.0% (4); Psychotherapist 28% (7); Supervisor (other prof title) 4% (1). Professional memberships: British Association for Counselling & Psychotherapy 16% (4); British Psychological Society 12.0% (3); Complimentary and Natural Healthcare Council 32.0% (8); General Medical Council 16.0% (4); National Hypnotherapy Society 4.0 (1); UKCP 8.0% (2); Other prof memberships = 72% (18); AfSFH, Association of Neurolinguistic Programmers, General Hypnotherapy Register, NCS, APHP, (Association of Professional Hypnotists and Psychotherapists)*;* BAThH (British Association of Hypnotists & NLP practitioners; General Hypnotherapy Standards Council (GHSC); General Hypnotherapy Register (GHR). National Counselling Society (NCS). There were no expert witnesses in this group.

Clinical psychologists (excluding EMDR practitioners) (*n* = 24); *M*_age_ = 51.29; *SD* = 12.7; range = 50 [26–76]. Biological sex, female = 58.3% (14); male 41.7% (10). Practice type: NHS 66.7% (16); private sector, 12 (50.0%). Other professional titles: One participant selected “other” psychologist, but gave no further detail. Professional memberships: BPS 22 91.7%; EMDRUK 25% (6); Other, 13 (54.2%): Association of Clinical Psychologists (ACAT); Health & Care Professional Council & British Association of Behavioural and Cognitive Psychotherapies.

***Trauma-focused mental health professionals***. Internal Family Systems’ (IFS) therapists, (*n* = 15), *M*_age_ = 53.2; *SD* = 9.29; Range = 32 [36–68]; Biological sex: females 12 (80%), males 3 (20%). Practice type: NHS 0.0%, private practice *n* =15, (100.0%). Other professional titles of IFS ticked, CBT therapist = 1 (6.7%); Counsellor = 7 (46.7%); Humanistic therapist 3 (20.0%); NLP, 1 (6.7%); Psychotherapist, 4 (26.7%); Trauma informed therapist, 4 (26.7%). Professional memberships were: Association of Christians in Counselling, 1 (6.7%); British Association for Counselling & Psychotherapy, 11 (73.3%); British Psychological Society 6.7% (1); UK Council for Psychotherapy, 2 (13.3%); Health & Care Professions Council, 6.7% (1). One participant was not registered with any professional body.

***The EMDR trauma focused group.*** Eye movement desensitisation and reprocessing practitioners (*N* = 62): *M*_age_ = 53.7, *SD* = 10.96, Range 46 [30-76]. Biological sex, females, 54, (87.1%) and males 8, (12.9%). Years of clinical experience, *M* = 20.21; *SD*, 9.99; Range 43 [2 – 45]. Practice type: NHS, 24 (38.7%), Private sector, 51 (82.3%). Online practice, 13 (21.0%). Professional titles are shown in these Supplemental Materials at Table S.4.

EMDR practitioners were registered with various professional and therapeutic organisations (many practitioners had multiple memberships). The most common memberships were: EMDR Association UK (EMDRUK) 74.2%, (*n* = 46), the British Association for Counselling and Psychotherapy (BACP) 35.5%, (*n* = 22), the British Psychological Society (BPS) 16.1%, (*n* = 10), British Association of Behavioural and Cognitive Psychotherapies (BABCP) 16.0%, (*n* = 10). Other professional memberships were: British Association for Counselling & Psychotherapy (BACP) 35.5% (22); Complimentary and Natural Healthcare Council (CNHC) 3.2% (2); Counselling and Psychotherapy in Scotland (COSCA) 1.6% (1); EMDR UK 74.2% (46); General Medical Council 4.8% (3); National Hypnotherapy Society 1.6% (1); Royal College of Psychiatrists 3.2% (2); UK Council for Psychotherapy 9.7% (6). Other (unlisted) professional memberships were added by 58.1% (36) participants, these were: Association of Clinical Psychologists UK 1.6% (1); Association of Core Process Psychotherapy, 1.6% (1); British Association of Behavioural and Cognitive Psychotherapies (BABCP)16.0% (10); British Psychoanalytic Council 1.6% (1); Dyadic Developmental Psychotherapy (DDP) 1.6% (1); Accredited Counsellors, Coaches, Psychotherapists and Hypnotherapists, (FACCPH), British Association of Therapeutic Hypnotists & Neurolinguistic Programmers (BAThH), General Hypnotherapy Register (GHR), General Hypnotherapy Standards Council (GHSC) 1.6% (1) (this one participant was a member of all the preceding organisations); General Medical Council 4.8% (3); Health and Care Professions Council (HCPC) 12.8% (8); International Centre for Excellence in Emotionally Focused Therapy (ICEEFT of EFT) 1.6% (1); Interpersonal Psychotherapy UK (IPTUK) 1.6% (1); National Hypnotherapy Society 1.6% (1); Nursing and Midwifery Council (NMC) 6.4% (4); Royal College of Occupational Therapists 1.6% (1); Social Work England 4.8% (3);

Six clinical psychologists and three psychotherapists assigned to the non-trauma-focused study group also ticked the box for membership of EMDRUK (the largest training body for EMDR practitioners). However, these participants did not tick the professional title ‘EMDR practitioner’. A condition of EMDRUK membership is completion of an EMDR training course. Therefore, whilst these survey participants may have practised EMDR it could not be assumed that they were currently practising. Consequently, to retain study these nine survey participants were treated as non-trauma-focused therapists, and specifically, not as EMDR practitioners.

**Materials**

**Table S.5** The Memory Belief Questionnaire

| *Instructions to participants:*  To what extent do you agree with the following statements? | Source |
| --- | --- |
| Item (1)  The mind is like a computer, accurately recording events as they actually occurred^a^ | Yapko et al.  (1994) |
| Item (2)  I believe that early memories, even from the first year of life, are accurately stored and retrievable | Yapko et al.  (1994) |
| Item (3)  Memory is constantly being reconstructed and changed every time we remember something^b^ | Patihis et al.  (2014) |
| Item (4)  The memory of everything we’ve experienced is stored permanently in our brains, even if we can’t access all of it | Patihis et al.  (2014) |
| Item (5)  Traumatic memories are often repressed (which means the person cannot remember the traumatic event due to a defence against painful content | Patihis et al. (2014) |
| Item (6)  Repressed memories can be retrieved in therapy accurately | Patihis et al. (2014) |
| Item (7)  Hypnosis can accurately retrieve memories that previously were not known to the person | Patihis et al. (2014) |
| Item (8)  It is possible to develop false memories for abuse/trauma that did not happen^c^ | Kemp et al. (2013) |
| Item (9)  Dissociative amnesia prevents a person from recalling traumatic experiences^b^ | New |
| Item (10)  What is/are your knowledge source(s) for your answers to the above questions: professional education; private reading; social media; TV documentaries and/or films and/or innate beliefs; Other (a text option was available for participants’comments.. | New |
| *Note*: ^a^ Items (1) and (2) Likert scale: 1 = D*isagree* *strongly*; 2 = *Disagree slightly*; 3 = *Agree* *slightly* ; 4 = *Agree strongly. ^b^* Items (3) to (7) and (9) Likert scale: 1 = *Strongly disagree*; 2 = *Disagree*; 3 = *Slightly disagree*; 4 = *Slightly agree*; 5 = *Agree*; 6 = S*trongly agree*. ^c^ Item 8 Likert scale: 1 = *Disagree*; 2 = *Somewhat disagree*; 3 = *Don’t know*; 4 = *Somewhat agree*; 5 *= Agree*. | |

***Mental Health Professionals’ Survey Section 3***

Four questions replicated previous survey items, (Pope et al. 1999) namely: Q1. ‘*If the DSM-5 were to be revised today, how should it treat the diagnosis of dissociative amnesia?*’ Q2. *‘If the DSM-5 were to be revised today, how should it treat the diagnosis of dissociative identity disorder?’* The answer choices (using a single tick box response) for both questions were as follows: (i) Should not be included at all; (ii) Should be included only with reservations (e.g., only as a proposed diagnosis’); (iii) Should be included without reservations; (iv) No opinion. Next, Q3. *‘In your opinion, what is the status of scientific evidence regarding the validity of dissociative amnesia?’* Q4. *‘In your opinion, what is the status of scientific evidence regarding the validity of dissociative identity disorder?’* The answer choices (using a single tick box response) for both Q3 and 4 were as follows: (i) Little or no evidence of validity; (ii) Partial evidence of validity; (iii) Strong evidence of validity; (iv) No opinion. Next, two new research questions followed, *‘How many cases have you dealt with that met the criteria for a diagnosis of dissociative disorder since 2010’* and *‘How many cases of satanic ritualistic abuse have you dealt with since 2010?’* A numerical response was required for both questions. Two final questions replicated (Kemp et al. 2013): First, *‘Memories of past trauma that are inaccessible to conscious memory or ‘blocked out’ can cause an alteration in a person’s consciousness/dissociation and result in physical symptoms such as altered motor function or non-epileptic seizures.’* Answers were on a five point Likert scale: (1) Disagree; (2) Somewhat disagree; (3) Don’t know; (4) Somewhat agree; (5) Agree. Second, *‘In your opinion, what degree of risk does childhood sexual abuse present for developing medically unexplained symptoms?’* Answer choices were: (1)None; (2) Minimal; (3) Small; (4) Medium; (5) High; (6) Don’t know.

**Results**

**Table S.6** Degrees of Agreement for MBQ Items 3, 4, 5, 6, 7 and 9 by Study Group

|  | % (*n*) Degree of agreement | | | |
| --- | --- | --- | --- | --- |
| Likert scales of agreement | Non trauma-  focused  *n* = 92 | Trauma-  Focused  (Other)  *n* = 24 | Trauma-focused EMDR  practitioners  *n* = 62 | Experts ^a^  *n* = 10 |
| Item 3 | Memory is constantly being reconstructed and changed every time we remember something | | | |
| Slightly agree % (*n*) | 21.7 (20) | 33.3 (8) | 19.4 (12) | 0.0 |
| Agree % (*n*) | 45.7 (42) | 54.2 (13) | 48.4 (30) | 20.0 (2) |
| Strongly agree % (*n*) | 26.1 (24) | 8.30 (2) | 29.0 (18) | 60.0 (6) |
| Item 4 | The memory of everything we’ve experienced is stored permanently in our brains, even if we can’t access all of it | | | |
| Slightly agree % (*n*) | 25.0 (23) | 25.0 (6) | 21.0 (13) | 20.0 (2) |
| Agree % (*n*) | 21.7 (20) | 20.8 (5) | 14.5 (9) | 0.0 |
| Strongly agree % (*n*) | 6.50 (6) | 8.30 (2) | 14.5 (9) | 0.0 |
| Item 5 | Traumatic memories are often repressed (which means the person cannot remember the traumatic event due to a defence against painful content) | | | |
| Slightly agree % (*n*) | 24.2 (22) | 16.7 (4) | 33.9 (21) | 0.0 |
| Agree % (*n*) | 37.4 (34) | 37.5 (9) | 24.2 (15) | 20.0 (2) |
| Strongly agree % (*n*) | 16.5 (15) | 37.5 (9) | 24.2 (15) | 30.0 (3) |
| Item 6 | Repressed memories can be retrieved in therapy accurately | | | |
| Slightly agree % (*n*) | 38.5 (35) | 41.7 (10) | 24.2 (15) | 10.0 (1) |
| Agree % (*n*) | 12.1 (11) | 29.2 (7) | 25.8 (16) | 10.0 (1) |
| Strongly agree % (*n*) | 6.6 (6) | 4.20 (1) | 6.50 (4) | 0.0 |
| Item 7 | Hypnosis can accurately retrieve memories that previously were not known to the person | | | |
| Slightly agree % (*n*) | 31.8 (28) | 40.0 (8) | 22.6 (14) | 20.0 (2) |
| Agree % (*n*) | 15.9 (14) | 15.0 (3) | 12.9 (8) | 20.0 (2) |
| Strongly agree % (*n*) | 4.50 (4) | 0.0 | 3.20 (2) | 0.0 |
| Item 9 | Dissociative amnesia prevents a person from recalling traumatic experiences | | | |
| Slightly agree % (*n*) | 32.2 (29) | 25.0 (6) | 24.2 (15) | 20.0 (2) |
| Agree % (*n*) | 43.3 (39) | 50.0 (12) | 43.5 (27) | 50.0 (5) |
| Strongly agree % (*n*) | 8.9 (8) | 20.8 (5) | 22.6 (14) | 10.0 (1) |

*Note*: Participant groups: The *Non* trauma-focused group, (included the ten experts) comprised all mental health participants *except* Eye Movement Desensitization and Reprocessing (EMDR) practitioners, Internal Family Systems therapists, trauma informed therapists, and dissociative disorder specialists. The trauma-focused (other) group, comprised study participants excluded from the non trauma-focused group, *except* EMDR practitioners. The Trauma-focused EMDR group comprised EMDR practitioners only. ^a^The far right-hand column shows the individual agreement levels of 10 experts; all experts were study participants in the non trauma-focused group.

**Table S.7** Sources of knowledge for memory beliefs by group

| Group |  | Professional  Education | Private  Reading | Social  Media | TV^a^  and/or films | Innate  Beliefs |
| --- | --- | --- | --- | --- | --- | --- |
|  | *n* | % (*n*) | % (*n*) | % (*n*) | % (*n*) | % (*n*) |
| Lay Public | 419 | 16.0 (67) | 49.2 (206) | 17.7 (74) | 46.8 (196) | 62.8 (263) |
| Non-trauma  -focused | 92 | 90.2 (83) | 81.5 (75) | 3.3 (3) | 17.4 (16) | 21.7 (20) |
| Trauma-  Focused  (Other) | 24 | 100.0 (24) | 83.3 (20) | 4.2 (1) | 8.3 (2) | 33.3 (8) |
| Trauma-focused  EMDR | 62 | 95.2 (59) | 77.4 (48) | 1.6 (1) | 11.3 (7) | 19.4 (12) |
| *Note.* Participant groups: Lay public; The non-trauma-focused group, comprised all mental health participants *except* Eye Movement Desensitization and Reprocessing (EMDR) practitioners, Internal Family Systems therapists, trauma informed therapists, and dissociative disorder specialists. The trauma-focused group comprised study participants excluded from the non-trauma-focused group, *except* EMDR practitioners. The EMDR group comprised EMDR practitioners only. | | | | | | |

*.*

**Examples of participants’ further comments on sources of knowledge**

In the Non-trauma-focused group: “Buddhist psychology studies” and “Witnessing a client access parts of an event she previously had missing fragments of during EMDR. Of course, I can't know what actually happened during the event at the time, but the new memories were very plausible.” This comment suggests the participant was an EMDR practitioner, despite not selecting this title. In the trauma-focused group, one participant commented: “My own personal experience of my ‘body remembering’ being sexually abused as a teen. My mind still has trouble with this, all (although?) the there are many clear indications from my past that this did indeed happen including concerned adults at the time.”

**Analysis of participants who identified as expert witnesses**

For item 1, “*The mind is like a computer*…” 80 % (8) chose “Disagree strongly” and 20 % (2) chose “Agree slightly”. For item 2, “…*early memories, even from the first year of life are accurately stored and retrievable*…” 80 % (8) chose “Disagree strongly”, 10% (1) chose “Disagree slightly” and 10 % (1) “Agree slightly”. For item 3, “*Memory is constantly being reconstructed*…” 10 % (1) chose “Disagree strongly” and 10% (1) “Disagree slightly”. Two experts chose “Agree” and 6 (60%) “Strongly agree”. For item 4, “*The memory of everything we’ve experienced is stored permanently*…” 5 experts (50 %) chose “Strongly disagree”, 2 (20 %) chose “Disagree”, one “Slightly disagree” and two experts “Slightly agree”.

For item 5, “*Traumatic memories are often repressed*…”, 50 % disagreed to varying degrees, whilst 20% chose “Agree” (one psychiatrist and one clinical psychologist) and 30% “Strongly agree, (two non-clinical professionals and one psychiatrist).

For item 9, “Dissociative amnesia prevents a person from recalling traumatic experiences”. No expert chose “strongly disagree or slightly disagree” but two participants (one clinical psychologist and one psychiatrist) chose “disagree”. One psychiatrist and one clinical psychologist chose “Slightly agree” and five other experts chose “agree”. A Human Givens therapist chose “strongly agree”. Another expert psychiatrist/psychotherapist chose “Strongly disagree” for items 5, 6, and 7, but selected “Agree” for item 9, dissociative amnesia.

**Table S.8** Mental Health Participant Expert Witnesses’ responses to MBQ items, 5 through 9

| Professional title, nation, &  memberships | Practice  NHS/  Private | Age | Item 5  Repression | Items 6  Accurate  therap’tic  retrieval | Item 7  Accurate  hypnotic  retrieval | Item 8  False memo | Item 9  Dissociative  Amnesia |
| --- | --- | --- | --- | --- | --- | --- | --- |
| Human Givens (NI).Royal Coll. of Speech & Lang. therapist | Private  only | 55 | Strongly  Agree | Disagree | Agree | Agree | Strongly  Agree |
| Psychiatrist (E)  GMC/ RCPsych. | Private  only | 67 | Strongly  agree | Disagree | Disagree | Agree | Agree |
| Medical device  Trainer, (E) HCPC | NHS  only | 58 | Strongly  Agree | Agree | Agree | Don’t know | Agree |
| Clinical  Psychologist  (E /W) | NHS &  Private | 74 | Disagree | Strongly disagree | Strongly  disagree | Agree | Disagree |
| Psychiatrist (E)  GMC; RCPsych | NHS &  Private | 73 | Agree | Strongly  disagree | Strongly disagree | Agree | Slightly agree |
| Psychiatrist (E)  GMC; RCPsych;  AACN, APA | NHS &  Private | 54 | Slightly disagree | Strongly  disagree | Strongly  disagree | Agree | Disagree |
| Psychiatrist &  Psychotherapist (E) GMC;  British Psycho- analytical society | Private  only | 62 | Strongly  Disagree | Strongly  disagree | Strongly disagree | Agree | Agree |
| Clinical psychologist (E)  BPS | Private  only | 73 | Agree | Slightly agree | Slightly agree | Agree | Agree |
| Clinical psych. (E); BPS | Private  only | 57 | Disagree | Strongly  disagree | Strongly  disagree | Agree | Slightly  agree |
| Clinical psych  (E), BPS; BACP; EMDRUK | Private  only | 50 | Slightly  disagree | Slightly disagree/ | Slightly agree | Agree | Agree |
| *Note*. Nation key: NI, Northern Ireland; E, England; E/W England and Wales. Professional memberships: AACN, APA, BPS British Psychological Society; BACP, British Association of Counselling and Psychotherapy; GMC, General Medical Council; RCPsych, Royal College of Psychiatrists. | | | | | | | |

**Table S.9** Comparing Group Beliefs for the Diagnostic Inclusion of Dissociative Amnesia

|  |  | Item question:  If the DSM-5 were to be revised today,  how should it treat the diagnosis of dissociative amnesia? | | | |
| --- | --- | --- | --- | --- | --- |
| Participant group | *n* | Should not be included at all  % (*n*) | Include only with reservations ^b^  % (*n*) | Include without reservations  % (*n*) | No opinion  % (*n*) |
| Non-trauma-focused | 77 | 7.8 (6) | 32.5 (25) | 18.2 (14) | 41.6 (32) |
| Trauma-focused  EMDR therapists | 57 | 3.5 (2) | 22.8 (13) | 31.6 (18) | 42.1 (24) |
| Trauma-focused (Other) | 21 | 19.0 (4) | 38.1 (8) | 9.5 (2) | 33.3 (7) |
| American  Psychiatrists (1999)^a^ | 301 | 9.0 (27) | 48.0 (143) | 35.0 (104) | 9.0 (27) |
| *Note*: This table is adapted from, “Attitudes Toward DSM IV Dissociative Disorders Diagnoses Among Board-Certified American Psychiatrists” Pope et al., 1999, *American Journal of Psychiatry.* ^a^ Their study data – participants were Board certified psychiatrists - was included for comparison. ^b^ The full text of this  response was, “ *Should be included only with reservations, (e.g., only as a proposed diagnosis’)*.” Participant groups. Participant groups: Lay public; *Non* trauma-focused group, comprised all mental health participants *except* Eye Movement Desensitization and Reprocessing (EMDR) practitioners, Internal Family Systems therapists, trauma informed therapists, and dissociative disorder specialists. The trauma-focused, (Other) group, comprised study participants excluded from the non-trauma-focused group, *except* EMDR practitioners. The Trauma-focused EMDR group comprised EMDR practitioners only. The percentage of participants who responded from each group were: non trauma-focused group 83.7% (77); the trauma-focused group 87.5% (21); the EMDR group, 91.9% (57). | | | | | |

**Table S.10** Comparing Group Beliefs for the Scientific Validity of Dissociative Amnesia

|  | Item question:  In your opinion, what is the status of scientific evidence regarding the validity of dissociative amnesia? | | | | |
| --- | --- | --- | --- | --- | --- |
| Participant group | *n* | Little or no evidence of validity  % (*n*) | Partial evidence of validity  % (*n*) | Strong evidence of validity  % (*n*) | No opinion  % (*n*) |
| Non-trauma-focused | 78 | 2.6 (2) | 32.1 (25) | 16.7 (13) | 48.7 (38) |
| Trauma-focused  EMDR therapists | 57 | 1.8 (1) | 31.6 (18) | 24.6 (14) | 42.1 (24) |
| Trauma-focused (Other) | 21 | 14.3 (3) | 28.6 (6) | 14.3 (3) | 42.9 (9) |
| American  Psychiatrists^8^  (1999) | 301 | 19.0 (56) | 48.0 (145) | 23.0 (69) | 10.0 (31) |
| *Note*: This table is adapted from, “Attitudes Toward DSM IV Dissociative Disorders Diagnoses Among Board-Certified American Psychiatrists” Pope et al., 1999, *American Journal of Psychiatry.* Their study data - participants were Board certified psychiatrists - was included for comparison purposes. Participant groups are as explained in Table S.9 above. The percentage of participants who responded from each group were: Non-trauma-focused group 84.8% (78); the Trauma-focused (Other) group 87.5% (21); the EMDR group, 91.9% (57). | | | | | |

***Further detail for participants reporting seeing many cases of DID and SRA***

***In the Non-trauma focused group***. Four participants claimed seeing over > 25 cases of DID were: a female (aged 65), hypnotherapist and CBT therapist, (96, DID cases), a male (aged 65) hypnotherapist, psychotherapist and counsellor (40, DID cases); a female (aged 63) Human Givens practitioner and member of the National Hypnotherapy Society (30, DID cases) and a male (aged 57) whose professional title was described as a “trainer of clinical hypnotherapy” (25 DID cases). All four were registered with mainstream therapeutic regulatory bodies. The participant who claimed seeing 96 DID cases was aged 65, and identified as a female hypnotherapist, counsellor and cognitive behavioural therapist with 30 years clinical experience. She was registered with the BACP, General Medical Council and UKCP. She “agreed slightly” that the mind works like a computer (MBQ item 1), “disagreed slightly” that early memories are accurately stored (item 2) and “agreed” that memory is constantly being reconstructed (item 3). However, she “slightly agreed” traumatic memories are often repressed (item 5) and “strongly agreed” that repressed memories can be retrieved in therapy accurately (item 6). She “slightly disagreed” that hypnosis can accurately retrieve memories (item 7) and “slightly disagreed” that dissociative amnesia prevents a person from recalling traumatic experiences (item 9). For item 8, false memories, she chose “don’t know”. For Section 3 questions concerning the scientific and diagnostic validity of dissociative amnesia and DID, this participant chose the “No opinion” response. This participant “somewhat agreed” blocked out memories of trauma can result in altered motor function and that child sexual abuse posed a “medium” risk of medically unexplained symptoms. This participant had also dealt with the highest number of cases of satanic ritual abuse (*n* = 9).

***Within the Trauma focused EMDR practitioner group*.** Two outlier participants claimed seeing 30 cases of DID. One was a female practitioner aged 61 with a mixed NHS and private practice. Her professional titles were, cognitive behavioural therapist, trainer, counsellor, hypnotherapist, psychologist (“other”), trauma-informed therapist, neurologist and neurolinguistic programmer. She was registered with various mainstream professional bodies, including the National Hypnotherapy Society, but not “EMDRUK”. She chose “strongly agree” for the following MBQ items: 1, the mind works like a computer; item 3, memory is being constantly reconstructed; item 6, accurate therapeutic retrieval of repressed memories; item 7 accurate hypnotic memory retrieval and item 9 (dissociative amnesia). She chose “agree” for item 5, traumatic repression and “disagree” for item 8, that false memories are possible. She also endorsed (without reservation) the diagnostic inclusion of DID and dissociative amnesia and DID. Furthermore, she believed there was strong evidence for the scientific validity of DID.

Another female EMDR practitioner aged 43, had also seen 30 DID cases. She identified as a clinical psychologist, Internal Family Systems and trauma-informed therapist and member of EMDRUK. She chose “disagree strongly” for MBQ item 1, the mind works like a computer, “disagree” for item 7, accurate hypnotic retrieval) and “somewhat disagree” for item 8 that false memories are possible. She chose “strongly agree” for MBQs items 5, traumatic repression and 9, dissociative amnesia, and “agree” for item 6, accurate therapeutic memory retrieval. Neither of these two EMDR participants had handled a case of satanic ritual abuse.

**Table S.11** Mental Health Professionals’ Optional Further Comments and MBQ beliefs for Items 5 & 9

*Key*: CBT, Cognitive Behavioural Therapist; EMDR, Eye movement desensitization and reprocessing; IFS, Internal Family Systems therapist; NHS, National Health Service: NLP, Neurolinguistic programmer; Online, online practice; PP, Private Practice; TIT, Trauma informed therapist.

| Professional  title | Optional Further comments | Belief in  traumatic  repression | Belief in  dissociative  amnesia |
| --- | --- | --- | --- |
| Hypnotherapist;  NLP^1^.  PP^2^ & Online^3^ | We record everything but don't remember accurately. I'm not sure we're meant to recall trauma, what purpose does that serve? We need to deal with the legacy, the results, not the event. We can't change the event | Strongly  Agree (6) | Strongly  Agree (6) |
| Counsellor;  Hypnotherapist.  NHS^4^ & PP. | Alcohol or substances impact how the memory works and codes data. | Agree (5) | Strongly  Agree (6) |
| Hypnotherapist;  NLP;TIT^5^.  PP. | The missing part here is that at the time of making a memory it is ‘accurate’ according to the meaning we make in the moment. It is always filtered through our existing beliefs, and can never be ‘the truth’. The difficulty with calling memories ‘false’ is that it opens a huge scope for disbelieving victims, and for them not receiving justice for that which was done to them. Science has not yet uncovered the truth about memory, consciousness and thought so it’s fascinating and a very broad topic. My main aim when working therapeutically is to ensure people are well resourced in the here and now, so that they can make the best sense of what’s happened to them and move on with a happy fulfilling life. | Strongly  Agree (6) | Agree  (5) |
| CBT^6^; Hypnotherapist.  PP. | I think we do store all our memories, but they can be altered when we retell them to ourselves, as well as externally and then that becomes embedded as a new memory. I think people can repress memories, but in most cases though not all they can be retrieved. Rather like memory alterations, I believe people can tell themselves a story about an event that did not happen and make it their reality to justify their behaviour or beliefs.  I have read widely and researched the subject, I have trauma within my family which has given me close up experience, as well as dealing with trauma and memory with my clients. | Strongly  Agree (6) | Agree (5) |
| Hypnotherapist;  PP. | Seeing and learning first hand from clients and from ‘live’ training just how much detail - when done correctly - hypnotherapy can create in clients | Agree (5) | Slightly  Agree (4) |
| Hypnotherapist;  NLP. PP. | Life experience | Agree (5) | Disagree  (2) |
| NLP.  PP & Online. | I have difficulty with the word ‘accurate’ to describe memory - it’s not possible to know whether a recollection is objectively ‘accurate’ or not. It is simply the recollection of something that a person is able to bring to mind at a particular moment on time. No third party is able to judge whether or not it is or is not ‘accurate’. | Slightly  Agree (4) | Agree (5) |
| CBT; TIT.  Counsellor,  EMDR^7^; NLP;  Hypnotherapist;  ‘Neurologist’;  Psychologist;  National Hypnotherapy Society; COSCA;  NHS & PP. | Having started as a therapist, counsellor I have progressed to trainer, which has the advantage of learning from progressive students of other perspectives/experiences through their journey, these learnings have meant hands on is vital to get a working model to excel in performance. Such has also assisted me in gathering information to become an inventor of the first inventor of cognitive synaptic cap medical device now on the nhs & worldwide | Agree (5) | Strongly  Agree (6) |
| Hypnotherapist  (BPS registered  Psychologist)  PP. | Memories can be altered with recall especially in a shared memory setting. Sometimes trauma can suppress memories; other times it can cause the thought to overwhelm. | Agree (5) | Agree (5) |
| CBT;  EMDR therapist;  Psychologist;  PP. | qu 2 is two questions. I think they are accurately stored but not always retrievable as they may be stored somatically. qu 4, I know nothing about hypnotherapy so a 'don't know' option would have been more accurate | Agree (5) | Agree (5) |
| Human Givens;  Psychotherapist.  PP | Traumatic memories can be repressed and can be restored Any recall experience is the most recent version of the event.  Our memories are affected in some way by our experiences and other external input Early pre verbal experiences can be retrieved and verbalised later especially if triggered by a sensory experience | Agree (5) | Strongly agree (6) |
| No response for professional title but BACP & EMDRUK membership.  NHS. | I have witnessed in the course of EMDR work people recovering memories and body held sensations | Strongly agree (6) | Strongly  Agree (6) |
| Psychotherapist  PP. Aged 74yrs | Meditation - see memories and perceptions arising in the moment | No  response | No response |
| Humanistic;  IFS^8^;  Psychotherapist.  PP | Your question about memories being stored accurately in our brains is interesting as I make a distinction between our mind and our brain. There is a connection between them. I think that memory lives in our minds, in relationship to neural connections in the brain. You also so not mention the meaning making that people form in response to events, including traumatic events. So, the questions are interesting. 'Memory is being reconstructed each time we remember something' I would add here that during therapy there is the  possibility of a radical reconstruction as opposed to a real of event in conversation. | Agree (5) | Agree (5) |
| Counsellor  IFS;  NLP. PP. | Dissociative amnesia initially blocked access to traumatic memory, but over time ( years in my case) traumatic memories were accessed and worked through in therapy. | Strongly  Agree (6) | Slightly  Disagree (3) |
| CBT; EMDR;  Hypnotherapist;  IFS;  Mental Health  Nurse; TIT.  PP. Online.  (Note: Not  Member of EMDRUK) | Factual accuracy is not important to me as a therapist so much as the felt sense, and the meaning that the client gives to their experience. Memories may be more like dreams, and it is the emphasis and symbolism that is to be taken notice of. If 20 people have the same experience, exactly, there will still be 20 completely different accounts of it. I don’t know why this is particularly applied to things that historic sex abuse it will be just the same with a burglary. I guess the difference is there will be other evidence also for the burglary where is in cases of historic sexual abuse is one persons word against another as a rule. | Agree (5) | Strongly  Agree (6) |
| CBT; Counsellor;  Humanistic;  IFS. PP | Everything is open to interpretation/memory/perception, so there cannot be definitive answers to these questions | Strongly agree (6) | Agree (5) |
| IFS. | Memories are not always accurate because they depend on the capacity of the person at the time experiencing them to process them properly. Children don't always remember things accurately because they can be laden with beliefs, for example. We can also aggregate memories or lose details - we are not a computer. Ultimately whether a memory is "factually accurate" is less important than what the memory contains for the client | Slightly agree (4) | Agree (5) |
| EMDR; IFS;  Psychotherapist;  TIT; Dissociative  Disorder specialist.  EMDRUK. PP. | Dislike word brain is a computer so cannot agree totally | Strongly agree (6) | Strongly agree (6) |
| CBT; EMDR;  Psychotherapist;  TIT. NHS only. EMDRUK; BABCP | Qu 5: the word often is problematic. Trauma memories can be repressed but I wouldn’t us the word often. I still believe this is relatively infrequent happening in the most difficult cases. | Slightly agree (4) | Agree (5) |
| Psychological therapist,  counsellor;  IFS; Psychotherapist;  PP. Online. | It would have been more accurate had the option of answering "I don't know" were available to other questions, not just one. I would have also answered "I don't know" to the following questions: "Traumatic memories are often repressed..."; "Hypnosis can accurately retrieve memories...";and  "Dissociative amnesia prevents a person from recalling traumatic experiences". | Slightly disagree (3) | Slightly  agree (4) |
| IFS.  PP. Online. | It is my understanding that in the view of the Internal Family Systems therapeutic model, memories are held differently by different parts of the individual's psyche and indeed, by their core Self. As we develop, mature and grow older, Self energy evolves a working memory congruous with life's everyday demands. In cases where an individual has been subjected to trauma, certain of their 'protector' parts will be called upon to comfort, numb, divert or distract the individual from the traumatic memories and associated feelings; an example of one such part might be dubbed 'dissociate amnesia.' In individuals where trauma has occurred, an accurate memory of what happened typically will be held by the wounded part(s) or exile(s) affected. In my experience, the exile's first recollection of the traumatic event is likely to be essentially accurate. Typically, this initial recollection and possible expression of the traumatic memory would happen during the 'witnessing' stage of the larger IFS protocol of witnessing, unburdening and retrieval. Subsequent recollection is likely to be affected by the tendency for memories to be reconstructed and changed every time we remember something, as identified above. | Agree (5) | Slightly  agree (4) |
| Humanistic;  IFS; TIT.  PP. | I've no experience of hypnosis | Strongly agree (6) | Strongly  Agree (6) |
| Psychotherapist.  PP. | I don't know enough about hypnotism to answer the question. | Slightly agree (4) | Slightly disagree (3) |
| Counsellor;  IFS; Psychotherapist;  TIT.  PP. | Memory is based on perspective, not absolute truth, and so whilst a memory can be real and true, is does not necessarily accurately reflect what actually happen. (My) therapy works with the client's memories as this is their LIVED truth where trauma etc resides. I don’t have any experience/knowledge of hypnosis, so cannot answer that question | Strongly agree (6) | Slightly agree (4) |
| CBT; EMDR;  Psychotherapist;  TIT.NHS & PP.  EMDRUK | Hard to answer as are quite absolute questions. | Slightly agree (4) | Slightly agree (4) |
| CBT; EMDR;  Psychologist.  PP. | The mind is evidently not like a computer. I don't have experience or sufficient knowledge of "false" memories to answer that question, but it is an immensely complicated area. When carrying out trauma processing in therapy, related memories and details do return to clients, how accurate they are is difficult to say and not necessarily relevant if relief from distress is achieved. Human memory tends to be inaccurate. | Slightly agree (4) | Slightly  Agree (4) |
| Counsellor;  Psychotherapist.  PP | A few problems with the questions - I have no opinion about hypnosis because it's not my field. Memories aren't necessarily stored in the brain - I believe the strongest evidence at the moment is that we do not know where memory is stored so that question contains an intrinsic bias. The idea of 'accurate' memory is problematic - the thing is as we access memories again our perspective changes because we are more able to understand the context in which they were formed - so the story changes, not the actual memory - a child's belief that 'I am not enough' can convert into an understanding that 'my mother didn't have enough to give me what I needed' and thus lose an unhelpful self-concept - the memory of what happened doesn't need to change, it is the understanding and belief about self that shifts | Agree (5) | Slightly  Agree (4) |
| EMDR; IFS;  Clinical Psychologist.  PP. | "Can" in some of the questions above is a complicating factor.  Yes, memories CAN be retrieved accurately in therapy, but not always or even most of the time, because they are subjective.  Accurate perhaps in the sense of what was encoded for the person at the time, but this may not match the objective reality of the external events, or someone else's encoded experience of the same event. | Slightly agree (4) | Slightly  Agree (4) |
| IFS.  PP.  BACP. | I’m not sure that I would use the wording above. Repressed suggests an element of choice which I don’t believe the client has with trauma. I also don’t know anything about hypnotherapy so can’t comment but don’t have a ‘don’t know’ option | Agree (5) | Strongly agree (6) |
| Counsellor;  IFS. PP. BPS – psychologist). | I have never recovered a lost memory, however I have recovered forgotten memories | Slightly disagree (3) | Agree (5) |
| EMDR; Mental health nurse;  Psychotherapist.  PP. EMDRUK. | Who determines the accuracy of early memories?  Early events are remembered, through the lens of the person involved,  preverbal events are stored as body sensations.  Memories can be fluid  - memory storage is fluid, memories are stored by association.  Memories are not fake but can be misinterpreted - for example a child waking from sleep under attack believes they are attacked by the devil - it transpires the devil is an individual in a halloween devil mask. | Slightly agree (4) | Agree (5) |
| Psychiatrist.  NHS & PP.  Expert witness | Memory is malleable | Slightly disagree (3) | Disagree (2) |
| Psychiatrist;  Psychotherapist.  PP. Expert wit‘s. | Memory is complex and can be affected by multiple factors including genetics; early experiences and influence by others. Both registration and recall can be problematic. | Strongly disagree (1) | Agree (5) |
| EMDR; Hypnotherapist.  PP. | I find it difficult to answer absolutes as it depends on the individual.  When I have said I agree with something it is because that is the case for the majority of people but not necessarily all.  For instance, I believe that most people don't accurately memorise things like a computer but there are some people that do.  The question about everything we have experienced not being stored in our brain is very likely to be true with our current understanding but we could discover in the future that it actually is. | Agree (5) | Agree (5) |
| Hypnotherapist;  NLP. PP. Online. | As unique individuals  how we experience and respond to trauma is different for each person. Their is no specific answer to most of your questions. | Slightly agree (4) | Disagree (2) |
| Clinical psychologist.  PP | I think that there are occasions when traumatic memories are repressed, and when these repressed memories can be reconnected with. This is particularly evident during EMDR. But there are lots of other aspects and factors which mean that memory for traumatic events may not be present or accurate. The aim of therapy is not to "retrieve" the memory, but to change the person's relationship with the stories that they hold about themselves. | Slightly agree (4) | Agree (5) |
| Clinical psychologist.  NHS & PP. | I believe the memory can be a highly subjective and unreliable recorder of our experience. I do believe the mind can protect one from traumatic memories with dissociation and repression, but I also believe that false memories can be 'retrieved'. However, I also believe that it is not the factual occurrence of events which effects us the most, but our interpretation and experience of those events. | Slightly agree (4) | Agree (5) |
| EMDR; Clinical  Psychologist; Psychotherapist.  NHS & PP. | The word false is difficult to use to describe memory, especially in the context of trauma. I do believe memories are subjective and rooted in our experiences, culture and meaning making. | Slightly agree (4) | Agree (5) |
| Mental health nurse; EMDR & EMDRUK; NHS only | Memories are our interpretation of events that can be real or imagined, not necessary a recording of actual events | Agree (5) | Agree (5) |
| Hypnotherapist;  Psychologist; BPS, BABCP; EMDR, & EMDRUK.  PP only. | I believe all memories are stored but not always accurately. In a sense this doesn't matter when we work with memories as it is the belief about the memory that impacts the clients belief about their selves and the world which is important. So we can work with a memory that isn't fully accurate. Regarding false memories- I think these are very unlikely | Strongly  Agree (6) | Strongly  Agree (6) |
| EMDR & EMDRUK; Systemic family therapist, PP only | Regarding your statement saying that traumatic memories are often repressed, I have a problem with the idea that this often happen. I believe memories CAN and SOMETIMES are repressed. A person can also dissociate from events and sometimes they are not recorded or not recorded fully because of that. (Other knowledge source = “ life experience” | Slightly disagree (3) | Strongly agree (6) |
| Clinical psychologist;  NHS & PP.  BPS; HCPC; EMDRUK | Memory is much more complicated than 'accurate exact recall of what happened in conscious awareness '. With memories of events in the first years of life I think it's unlikely most people will have conscious memories but likely they do have implicit/body-based memories | Disagree  (2) | Agree (5) |
| Psychotherapist.  PP only. EMDRUK | There is a lot we don't know and can't conceptualise accurately. | Strongly  Agree (6) | Slightly  Agree (5) |
| Psychotherapist;  TIT; Dissociative disorders specialist; psychoanalytic psychotherapist  NCPS. | You haven't asked about the need to recall, process, and experience memories in the present, how these infirm the transference, and bring meaning. (inform?) | Agree  (5) | Agree (5) |
| Clinical psychologist  PP only. BPS; EMDRUK; BABCP. | Parts of memories may not be accessible at times but may return | Strongly disagree | Agree (5) |
| Neurologist,  Clinical psychologist. BPS. EMDRUK.  HCPC.  NHS only | I think this is a very difficult area - research shows both that memory is malleable and recall has a different flavour throughout the lifespan but we also know that some recollections, particularly those with a somatic quality, reflect really early memories and accessing these in EMDR can bring great relief. This suggests that the brain is not a computer and recall is often inaccurate at the same time that memories are repressed and false memories can be created. A complex picture | Disagree | Agree (5) |

**Discussion**

**Further detail on the AIP model**

The AIP is EMDR’s unique theory of memory (Shapiro, 1989a, b,; 2010; 2014, 2018). The AIP model theorizes that memories for severe experiences are sometimes stored in a dysfunctional, implicit (unconscious), unprocessed, form (Ad de Jongh, et al., 2010; Royal College of Psychiatrists, (2021,b) and need to be transferred into explicit form (Shapiro, 2018) - to improve current symptoms. In other words, EMDR aims to modify both *known* memories, and *unknown*, (implicit) memories. Arguably, this idea overlaps with the posttraumatic model (Dalenberg et al., 2012) and body memory theory (Van der Kolk, 1994). However, the notion of body memories, and transference from implicit (non-narrative memory) into explicit memory, is scientifically questionable (see McNally, 2003; 2005; 2007).

**Past international guidance and position statements for mental health professionals**

To safeguard public health, professional regulatory bodies cautioned psychologists and psychiatrists against using RMT and warned of potential iatrogenic risks (see American Psychiatric Association, 1993; American Psychological Association, 1994; Australian Psychological Society (1994); British Psychological Society, 1995; (UK) Brandon Report, 1998; Canadian Psychiatric Association (1996) and (UK) Royal College of Psychiatrists, 1997).

**References**

Brandon, S., Boakes, J., Glaser, D., & Green, R. (1998). Recovered memories of childhood sexual abuse: Implications for clinical practice. *The British Journal of Psychiatry*, *172*(4), 296-307. doi:10.1192/bjp.172.4.296

Canadian Psychiatric Association, (1996). *Position Statement: Adult Recovered Memories of Childhood Sexual Abuse*. Toronto: CPA.

Clinic for Dissociative Studies <https://clinicds.org.uk/>

Dalenberg, C. J., Brand, B. L., Gleaves, D. H*.,* Dorahy, M. J., Loewenstein, R. J., Cardena, E., ... & Spiegel, D. (2012). Evaluation of the evidence for the trauma and fantasy models of dissociation. *Psychological Bulletin*, *138*(3), 550. <https://doi.org/10.1037/a0027447>

European Society for Trauma and Dissociation (ESTD) <https://estd.org/>

McNally, R. J. (2003). *Remembering trauma*. Belknap Press of Harvard University Press.

McNally, R. J. (2005). Debunking myths about trauma and memory. *Canadian Journal of Psychiatry*, *50*(13), 817–822. <https://doi.org/10.1177/070674370505001302>

McNally, R. J. (2007). Betrayal trauma theory: A critical appraisal. *Memory*, *15*(3), 280-294. <https://doi.org/10.1080/09658210701256506>

Mind. (2023). Dissociation and dissociative disorders. <https://www.mind.org.uk/media/anbbckla/dissociation-and-dissociative-disorders-pdf>

Nathan, D. (2011). *Sybil exposed: The extraordinary story behind the famous multiple personality case*. Simon and Schuster.

National Health Service. August 2023. Dissociative Disorders <https://www.nhs.uk/mental-health/conditions/dissociative-disorders/>

Royal College of Psychiatrists (1997) Reported recovered memories of child sexual abuse. Recommendations for good practice and implications for training, continued professional development and research. *Psychiatric Bulletin*, 21, 663-665.

Royal College of Psychiatrists. A Competence Framework for Eye Movement Desensitisation and Reprocessing Therapy. (2021b). Domain 4. Knowledge of the Adaptive Information Processing (AIP) Model <https://www.rcpsych.ac.uk/docs/default-source/improving-care/nccmh/emdr-therapy-competence-framework/emdr-therapy-competence-framework-domain-4-knowledge-of-the-adaptive-information-processing-(aip)-model.pdf?sfvrsn=c74e8e34_2>

Shapiro, F. (1989a). Eye movement desensitization: A new treatment for posttraumatic stress disorder. *Journal of Behavior Therapy and Experimental Psychiatry*, **20**, 211–217.

Shapiro, F. (1989b). Efficacy of the eye movement desensitization procedure in the treatment of traumatic memories. *Journal of Traumatic Stress*, **2**, 199–223.

Shapiro, F. (2001). *Eye movement desensitization and reprocessing: Basic principles, protocols, and procedures* (2nd ed.). New York: Guilford Press

Shapiro, F. (2010). EMDR therapy: Adaptive information processing, clinical applications and research recommendations. *APA Division 56 Trauma Psychology Newsletter*, *5*,12–18.

Shapiro, F. (2014). The role of eye movement desensitization and reprocessing (EMDR) therapy in medicine: addressing the psychological and physical symptoms stemming from adverse life experiences. *The Permanente Journal*, *18*(1), 71. <http://dx.doi.org/10.7812/TPP/13-098>

Shapiro, F. (2018). *Eye movement desensitization and reprocessing:*Basic principles, protocols and procedures (3rd ed.). Guilford Press.

The Pottergate Centre – The centre for trauma and dissociation https://dissociation.co.uk/
